# A Modular Implementation to Handle and Benchmark Drift Correction for High-Density Extracellular Recordings

**DOI:** 10.1523/ENEURO.0229-23.2023

**Published:** 2024-02-09

**Authors:** Samuel Garcia, Charlie Windolf, Julien Boussard, Benjamin Dichter, Alessio P. Buccino, Pierre Yger

**Affiliations:** ^1^Centre de Recherche en Neuroscience de Lyon, CNRS, Lyon 69675, France; ^2^Columbia University, New York, New York 10027; ^3^CatalystNeuro, Benicia, California 94510; ^4^Allen Institute for Neural Dynamics, Seattle, Washington 98109; ^5^Institut de la Vision, Sorbonne Université, INSERM, Paris 75012, France; ^6^Lille Neurosciences & Cognition (lilNCog)—U1172 (INSERM, Lille), Univ Lille, Centre Hospitalier Universitaire de Lille, Lille 59800, France

**Keywords:** benchmark, drift, electrophysiology, ground-truth, neuropixel, spike sorting

## Abstract

High-density neural devices are now offering the possibility to record from neuronal populations in vivo at unprecedented scale. However, the mechanical drifts often observed in these recordings are currently a major issue for “spike sorting,” an essential analysis step to identify the activity of single neurons from extracellular signals. Although several strategies have been proposed to compensate for such drifts, the lack of proper benchmarks makes it hard to assess the quality and effectiveness of motion correction. In this paper, we present a benchmark study to precisely and quantitatively evaluate the performance of several state-of-the-art motion correction algorithms introduced in the literature. Using simulated recordings with induced drifts, we dissect the origins of the errors performed while applying a motion correction algorithm as a preprocessing step in the spike sorting pipeline. We show how important it is to properly estimate the positions of the neurons from extracellular traces in order to correctly estimate the probe motion, compare several interpolation procedures, and highlight what are the current limits for motion correction approaches.

## Significance Statement

High-density extracellular recordings allow experimentalists to get access to the spiking activity of large neuronal populations, via the procedure of spike sorting. However, it is widely known that spike sorters are affected by *drifts*, i.e., the fact that neurons move with respect to the recording electrodes. While several algorithms have been proposed to handle drifts, a systematic comparison on the performance of these algorithms is still lacking. In this contribution, we performed a large comparison study to benchmark and understand the limitations of state-of-the-art drift correction methods. Our results suggest that they all have some intrinsic limitations that should be taken into account by analysis pipelines.

## Introduction

Recording from increasingly larger neuronal populations is a crucial challenge to unravel how information is processed by the brain. The recent development of complementary metal-oxide semiconductor-based high-density multi-electrode arrays (HD-MEAs), both for in vitro (Berdondini et al., 2009; Frey et al., 2009) and in vivo applications, such as Neuropixels probes (Jun et al., 2017; Angotzi et al., 2019; Steinmetz et al., 2021), has dramatically boosted the yield of extracellular electrophysiology experiments. To maximize the return from these novel probes, the “spike sorting” step, i.e., the process which extracts single neuron activities from the recorded traces, has also undergone major advances both in algorithmic development (Lefebvre et al., 2016; Pachitariu et al., 2016; Chung et al., 2017; Yger et al., 2018; Lee et al., 2020; Magland et al., 2020; Steinmetz et al., 2021; Buccino et al., 2022; Pachitariu et al., 2023) and quantitative benchmarks (Buccino et al., 2020; Magland et al., 2020; Garcia et al., 2022).

**Figure 1.**
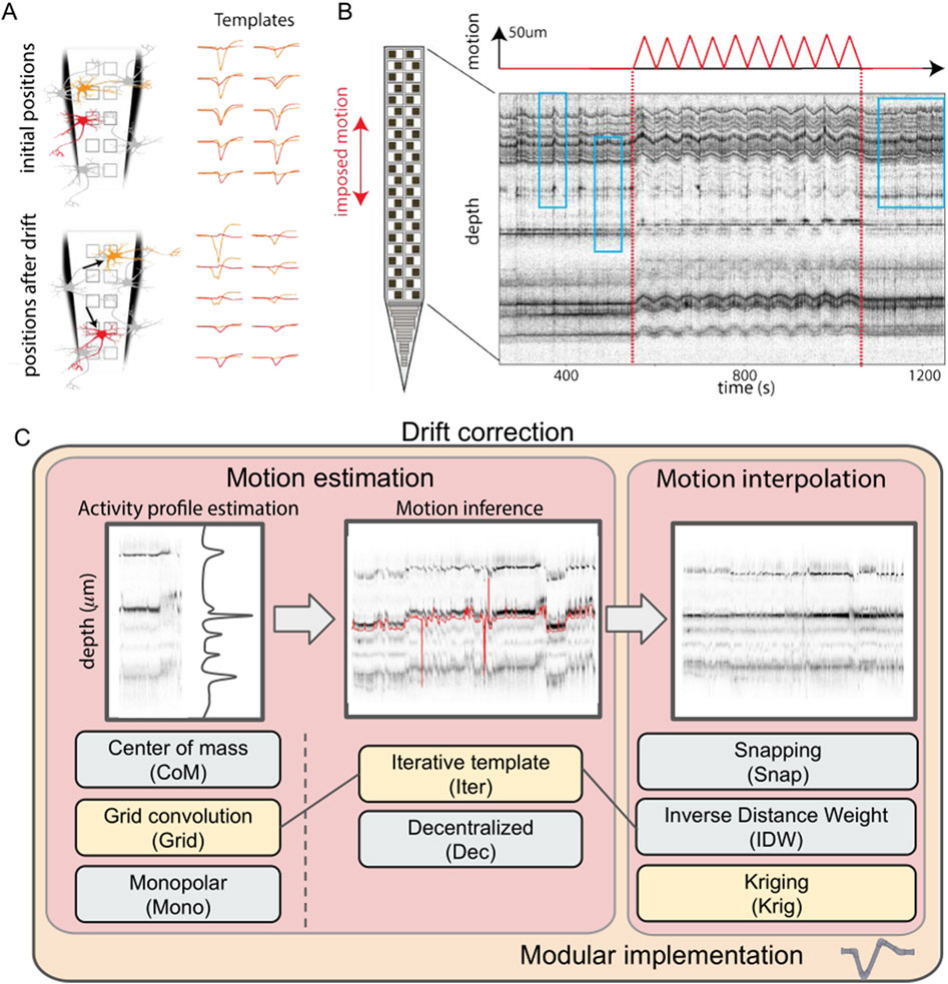
Common pipeline for drift correction algorithms. ***A***, Illustration of the problems raised by neuronal drifts. Two neurons (red and orange) have two distinct waveforms, but after some non-rigid drifts (movements), the templates are changed. ***B***, Illustration of the drift in vivo. A Neuropixels 1.0 probe in the cortex of the mice is moved up and down with an imposed triangular movement of amplitude 50 m (top trace; Steinmetz, 2021; Steinmetz et al., 2021)—p1 dataset. The row shows the depth of all the peaks detected *via* signal threshold (*positional* raster plot, see text) as a function of time. When the probe starts moving (red dotted line), so does the depth of the peaks, since neurons are drifting across channels. While the drift here is imposed, spontaneous drifts can happen locally and non-homogeneously over the whole probe (see inside light blue boxes for before and after imposed motion). Panels A and B are adapted from Buccino et al. (2022), with permission. ***C***, Key algorithmic steps for the motion correction pipeline. For each step, the various implementations available in SpikeInterface are listed. The yellow boxes connected with arrows correspond to a Kilosort 2.5/3-like approach (Pachitariu et al., 2023).

The layout of HD probes for in vivo applications usually consists of a long shank (e.g., ∼1 mm for Neuropixels 1.0; Jun et al., 2017) to allow the electrodes to span multiple regions of the brain and reach deep structures. Due to the different mechanical properties of the probe and the brain tissue, it is very common to observe a relative movement of the tissue with respect to the probe. This phenomenon is known as *drift*. The origins and types of such drifts can be diverse. For example, cells are likely to slowly drift from initial positions because of the pressure release in the tissue after an acute probe insertion; abrupt and discontinuous drift events could be caused by sudden rig instabilities and movement artifacts. When a neuron moves with respect to the recording electrodes, its waveforms are distorted (Fig. 1*A*), challenging the operation of spike sorting algorithms which mainly rely on waveform similarities to cluster different neurons in the recordings.

Due to the spatial regularities of the recordings, which makes drifts relatively coherent in space (Fig. 1*B* shows a *raster map* of a recording with an induced triangular probe movement; Steinmetz, 2021), a common approach is to mimic drift correction procedures that have been applied in the field of calcium imaging (Greenberg and Kerr, 2009). First, motion is estimated from the spiking activity extracted from the recording (Fig. 1*C*, left). Then, the estimate is used to interpolate or register the recording as a preprocessing step (Fig. 1*C*, right). Different methods have been recently proposed to correct drifts before spike sorting (Boussard et al., 2021; Steinmetz et al., 2021; Varol et al., 2021; Pachitariu et al., 2023), but their evaluation is mainly qualitative. A quantitative evaluation of the performance and effectiveness of different strategies is still lacking, probably due to the complexity of performing such benchmarks. To benchmark drift correction methods, one would need to know the ground-truth (GT) motion that is generating drifts. In addition, these methods are usually integrated into complicated spike sorting pipelines, and this makes it hard to track down what part of the error was really tied to the motion correction step.

In this work, we extensively benchmarked state-of-the-art methods for drift correction to solve the above-mentioned challenges. To obtain recordings with GT motion, we used biophysically detailed simulations with drifting neurons. This allowed us to systematically explore a wide range of drifting recordings of varying complexity. We further identified and broke down existing drift correction strategies into a modular architecture that allowed us to properly evaluate the performance of different methods for estimating motion, interpolating the traces, and their overall impact on spike sorting outcomes. All the source code used to generate the synthetic recordings and to reproduce results and figures (including Extended Data) is available at https://github.com/samuelgarcia/spike_sorting_motion_benchmark_paper_2023.

## Materials and Methods

### A modular implementation of drift correction

In order to properly evaluate and benchmark the latest in vivo drift correction methods available nowadays in the literature (Varol et al., 2021; Pachitariu et al., 2023), we first identified the individual sub-components of the different approaches and implemented a modular system to be able to substitute different steps. In fact, most of the available drift correction methods acting at the preprocessing level (Boussard et al., 2021; Pachitariu et al., 2023) share similar ideas, so that the global algorithmic pipeline can be decomposed and summarized as follows (Fig. 1*C*).

First, a sweep of spike detection (with a peak detection algorithm) is performed and the putative peak depths (defined as the position along probe’s insertion direction) for each action potential are estimated (this first step is usually done on a subset of peaks to speed up the computation time). The estimated depths are binned in short time windows (e.g., 2 s) to obtain an **activity profile** (Fig. 1*C*, left) as a function of depth. It is important to stress that a time window that is too small might not contain enough spikes to properly estimate the activity profile, while too large time windows might result in *blurred* activity profiles because of the drift within each time window. From the activity profiles varying in time, the **motion inference** step aims at recovering the motion signals (red line in Fig. 1*C*, middle). Finally, one can interpolate the traces with the negative motion signals to compensate for the estimated motion (**motion interpolation**, Fig. 1*C*, right).

The general pattern of these three processing stages is seen across different spike sorting pipelines, though the details and implementations of the different steps vary from algorithm to algorithm. For example, in Steinmetz et al. (2021) and Pachitariu et al. (2023), an average activity template is computed as the mean of activity histograms computed over all temporal windows and used as a reference for motion registration. Nevertheless, this *average template* strategy assumes a stationary spiking activity over the course of the recording. Recent works have proposed a decentralized method (Varol et al., 2021; Windolf et al., 2023) to relax this assumption, which uses all the pairwise displacements between activity profiles, instead of using a single reference template, to directly estimate a motion signal to register the traces.

We used the modular architecture of the sortingcomponents module of the SpikeInterface package (Buccino et al., 2020) to factorize and benchmark all the steps in a modular manner. As shown in the bottom of Figure 1*C*, we have implemented three major algorithms to estimate the positions of detected spike (see Methods), two methods to estimate the motion given these positions (the one from Kilosort , termed “íterative template,” and the one from Boussard et al., 2021 termed “decentralized,” see Methods), and three methods to interpolate the traces.

This modular implementation enables us first to benchmark the different drift correction steps in a sorter-agnostic manner. It is worth highlighting that these correction methods can be applied as a preprocessing step before any spike sorting algorithm. In addition, the modular implementation can also replicate existing drift correction strategies, including the one similar to Kilosort (since v2.5 (The implementation was ported from pyKilosort—https://github.com/int-brain-lab/pykilosort/); shown in yellow in Fig. 1*C*), and the motion estimation introduced in Boussard et al. (2021) and Varol et al. (2021) (note that in this case, the motion interpolation step was not used).

### Motion estimation

As illustrated in Figure 1*C*, motion estimation includes two distinct steps: activity profile estimation and motion inference.

### Notations

Throughout the article, vector variables are represented by the → notation. We use ${\vec{w}}_{i}(t)\in R^{N\times M}$ to represent the spatio-temporal waveforms emitted by the neuron *i* at time *t*, where *N* is the total number of channels and *M* is the number of time samples (by default, we cut out 2 ms signal at a sampling rate of 32 kHz, which makes *M* = 64). We further use the term GT to refer to the fully controlled variables in our synthetic recordings (either the motion signal or the spike times of the units).

#### Activity profile estimation (peak localization)

To estimate a histogram of the activity of the cells in a given time window *τ*_win_ (default is 2 s) as a function of depth, we used, in all cases, the estimated peak depths obtained via peak localization. We first detected peaks as negative threshold crossings with a spatio-temporal exclusion zone (see the “locally exclusive” method of SpikeInterface, with parameters detection_threshold=10, local_radius_um = 50 m, and exclude_sweep_ms=0.2ms). From the detected peaks, we estimated their depths in three ways:

##### Center of mass (CoM)

Assuming that the spike *i* has its waveform ${\vec{w}}_{i}(t)$ defined on several channels *c* ∈ {1, …, *N*}, we computed the peak-to-peak values ptp_*i*_(*c*) on every channel (also referred to as *peak-to-trough* amplitude). Since every channel has a physical position ${\vec{\;p}}_{c}=(x_{c}\;\;y_{c})$ in the 2D space, we can obtain, for every spike *i*, its CoM(*i*) as

$$\mathrm{CoM}(i)=\frac{\sum_{c}{\mathrm{ptp}}_{i}(c)\vec{\;p}(c)}{\sum_{c}{\mathrm{ptp}}_{i}(c)}.$$ (1)

##### Grid convolution (Grid)

Introduced in Pachitariu et al. (2023), the idea behind this localization method is to create an exhaustive catalog F of artificial templates ${\vec{\;f}}_{n\in 1\;\ldots \;k}(t)$ at known positions ${\vec{\;p}}_{n}=(x_{n}\;\;y_{n})$ and to estimate the position of a given waveform ${\vec{w}}_{i}(t)$ projected onto this catalog. If the spatial resolution of this grid is finer than the one of the recording channels, one could expect to enhance the resolution of the localization estimates. First, the scalar products $\omega_{i\;n}={\vec{w}}_{i}^{F}(t){\vec{\;f}}_{n}^{F}(t)$ of the waveform with all the templates in the catalog are computed; then, the spike position Grid(*i*) is estimated as a weighted sum of the positions, with weights equal to the scalar product between the waveform and these templates (negative scalar products are discarded):

$$\mathrm{Grid}(i)=\frac{\sum_{n}\omega_{i\;n}{\vec{\;p}}_{n}}{\sum_{n}\omega_{i\;n}}.$$ (2)

To create the artificial templates, the typical waveform $\vec{H}(t)$ of all detected peaks on a single channel is estimated as a median over 1,000 normalized waveforms, and then duplicated on all nearby channels, with a spatial decay in amplitude *σ* such that on every channel *c* at a position ${\vec{\;p}}_{c}=(x_{c}\;\;y_{c})$ we have:

$${\vec{\;f}}_{n}(c\;\;t)=e^{-({\vec{\;p}}_{c}-{\vec{\;p}}_{n}{)}^{2}/(2\sigma^{2})}\vec{H}(t).$$ (3)

To extend the catalog, one can use multiple *σ* values (in the range of 10–50 m). Moreover, in order to reduce the spreading of the scalar products if too many templates are in the catalog, we only perform the estimation in Equation 2 on the top 10% of the scalar product values.

##### Monopolar triangulation (Mono)

The idea of this method is to consider the cell as a monopolar current source (Buccino et al., 2018; Boussard et al., 2021) and infer its position by triangulation given the amplitudes of the waveforms recorded on all channels. Assuming the cell behaves as a monopolar current source, the extracellular peak-to-peak values ptp_*i*_(*c*) on a channel *c* generated by a neuron *i* at position ${\vec{\;p}}_{i}=(x_{i}\;\;y_{i}\;\;z_{i})$ can be expressed as

$${\mathrm{ptp}}_{i}(c)=\frac{k_{i}}{\sqrt{(x_{c}-x_{i}{)}^{2}+(y_{c}-y_{i}{)}^{2}+(z_{c}-z_{i}{)}^{2}}}$$ (4)

with the *k*_*i*_ term including the magnitude of the current and propagation properties of the tissue (see Buccino et al., 2018). Therefore, to estimate the location of the spike, one can simply solve an optimization problem with the cost function Φ(*x*, *y*, *z*, *k*) and find the (*x*_*i*_, *y*_*i*_, *z*_*i*_) estimated position (and (*k*_*i*_)) from the monopolar approximation by minimization:

$$\Phi (x\;\;y\;\;z\;\;k)=\sum_{c}{\left({\mathrm{ptp}}_{i}(c)-\frac{k}{\sqrt{(x_{c}-x{)}^{2}+(y_{c}-y{)}^{2}+(z_{c}-z{)}^{2}}}\right)}^{2}.$$ (5)

Such that the spike position Mono(*i*) is

$$\mathrm{Mono}(i)={\mathrm{argmin}}_{x\;y\;z\;k}\Phi (x\;\;y\;\;z\;\;k).$$ (6)

To minimize the cost function, we used the scipy.optimize (Virtanen et al., 2020) function with the Broyden–Fletcher–Goldfarb–Shanno algorithm.

#### Motion inference

From the activity profiles, the next step is to infer motion. Although the peak localization methods return 2D (CoM and Grid) and even 3D (Mono) locations, the motion inference step currently only uses the *y* coordinate, i.e., the depth of the probe. Therefore, the *y* estimates of the peak localization are also referred to as spike depths.

For this step, we considered two different methods to estimate the drift vectors, i.e., the putative drift at a given place in time and depth:

##### Iterative template (Iter)

The first method is the one implemented in Kilosort 2.5 (Steinmetz et al., 2021; Pachitariu et al., 2023). From the activity profiles, a 3D histogram is constructed using the spike counts, spike depths, and log-transformed spike amplitudes (i.e., the values of the trace when a putative spike is detected on the channel it was detected on) as dimensions. The spike counts are binned over 2 s temporal windows, depths using 5μm bins, and amplitudes in 20 equally distributed bins. Next, a rigid registration is computed across the time dimension by iteratively finding the shift that maximizes the correlation between each shifted temporal bin of the histogram and a target *average* template (maximum 15 shifts, each corresponding to one spatial bin of 5μm), which is also iteratively updated (for the first iteration, an activity profile sample at the center of the recording is used). The correlation is computed as the mean of the element-wise product between the shifted temporal bin and the average template. Next, the depth is divided into sub-blocks of 50μm to refine the estimation by taking into consideration non-rigid effects. For each block, a similar procedure, but without iteration, is performed to find the best alignment for each spatial block. All parameters were the same as the default parameters of Kilosort2.5. The only minor difference is that Kilosort2.5 defines the temporal bins in number of samples (default 65’600 ∼ 2.18 s), while we used 2 s.

##### Decentralized (Dec)

The second method is from Varol et al. (2021). First, it constructs a 2D motion histogram from the activity profile, by binning peak locations in time and depth, using 2 s temporal bins and 5 μm spatial bins. The main difference between this approach and the iterative template one is that the correlation is not computed with respect to a target average template, but, instead, a correlation value is computed between each pair of temporal bins (optionally, a time *horizon* can be used to limit the correlation computations to bins within a certain interval to speed up the computation, e.g., 120 s). This results in a pairwise correlation matrix, which is then used to estimate the motion that maximizes all pairwise correlations, using the LSMR solver (for details, refer to Varol et al., 2021). To achieve non-rigid motion estimation, the same approach is applied on spatial blocks of 50 μm and a spatial prior is used to enforce smoothness between consecutive spatial blocks (Windolf et al., 2023). Note that differently from the *Iter* method, where first a global inference is performed, followed by a non-rigid refinement, here the motion of each spatial block is inferred separately, and a final smoothing operation between blocks is applied.

### Motion interpolation

Once the drift vectors have been estimated, the next step of the pipeline is to interpolate the extracellular traces. Given all the values of the signals *s*_*c*_(*t*) at channels *c* at positions ${\vec{\;p}}_{c}=(x_{c}\;\;y_{c})$, the goal is to determine the value *s*(*t*) of the extracellular traces at a drift-corrected position $\vec{\;p}=(x\;\;y)$ which accounts for the estimated motion. Here again, several methods to interpolate the extracellular traces from the motion vector have been implemented. To be more efficient, interpolation kernels are computed at the temporal resolution of the time histograms used to compute the activity profiles in a chunk-wise manner, not at every time point.

#### Snapping (Snap)

In this case, given an estimated position $\vec{\;p}$, the value of the extracellular traces at the closest electrode from the drift vector is used:

$$s(t)=s_{c^{*}}(t)$$ (7)

with c* such that we have $\min_{c}\|\vec{\;p}-{\vec{\;p}}_{c}{\|}_{2}$.

#### Inverse distance weighting

The interpolated value is a weighted sum of the extracellular values on the channels in the vicinity of the estimated position $\vec{\;p}$, with weights determined by the distances:

$$s(t)=\frac{\sum_{c}w_{c}s_{c}(t)}{\sum_{c}w_{c}}$$ (8)

with $w_{c}=\|\vec{\;p}-{\vec{\;p}}_{c}{\|}_{2}$. In practice, this weighed sum is restricted to the three nearest channels.

#### Kriging (Krig)

Finally, we also implemented the kriging method, as used in Kilosort (Pachitariu et al., 2023). To be more precise, we computed *K*_*xx*_ as the negatively exponentiated distance matrix between all the positions of the known channels ${\vec{\;p}}_{c}$. To account for the different *x* and *y* scales of the Neuropixels layout, Kilosort uses different scaling factors *σ*_*x*_ and *σ*_*y*_, and we reuse the exact same formula for comparison purposes. However, a more generic framework has been implemented in SpikeInterface to account for the general distances. The distance considered for *K*_*xx*_ in this paper is thus a city-block distance between all channels *i*, *j* such that:

$$K_{xx}=e^{-|x_{i}-x_{j}|/\sigma_{x}-|y_{i}-y_{j}|/\sigma_{y}}.$$ (9)

In practice, we set $\sigma_{x}=20\;\mu \text{m}$ and $\sigma_{y}=30\;\mu \text{m}$. Then we computed *K*_*yx*_ also as the negatively exponentiated distance matrix between $\vec{\;p}$ and all the channels ${\vec{\;p}}_{c}$. The kriging kernel *W* is obtained as

$$W=K_{yx}\dot{(}K_{xx}+0.01\cdot I{)}^{-1}.$$ (10)

The kernel is sparsified so that all values below ϵ=0.001 are set to 0 and normalized so that each column of *W* sums up to 1.

### Simulated datasets

We used the MEArec simulator (Buccino and Einevoll, 2020; version 1.9.0) to generate 10-min-long synthetic GT recordings. MEArec uses biophysically detailed multicompartment models to simulate extracellular action potentials, or so-called “templates.” For this study, we used 13 *default* cell models from layer 5 of a juvenile rat somatosensory cortex (Markram et al., 2015; Ramaswamy et al., 2015) to simulate a dictionary of biologically plausible templates on a Neuropixels probe layout (cropped to 128 electrodes in 4 columns and hexagonal arrangement, a *x*- and *y*-pitch of 18 and 22 μm, respectively, and an electrode size of 12 μm per side). To simulate drift, the templates (100 for each cell model) were obtained by virtually moving the cell models along a 100 μm straight vertical line with 1 μm steps starting from random initial positions. For each recording, we then selected 256 neurons and generated corresponding spike trains. Templates and spike trains were then convolved following the drift signals and adding a slight modulation in amplitude to add physiological variability. Finally, uncorrelated Gaussian noise with 5μV standard deviation (STD) was added to the traces. The sampling rate of the simulated recordings was set to 32 kHz. Please refer to Figure 6 of Buccino and Einevoll (2020) for additional details on simulating drift.

To challenge the drift correction algorithms, we generated various drift recordings using different drift signals, depth distributions, and firing rate profiles. In doing so, we tried to capture the key features that could be observed in experimental recordings obtained in vivo, especially for Neuropixels probes.

#### Drift signals

**Figure 2.**
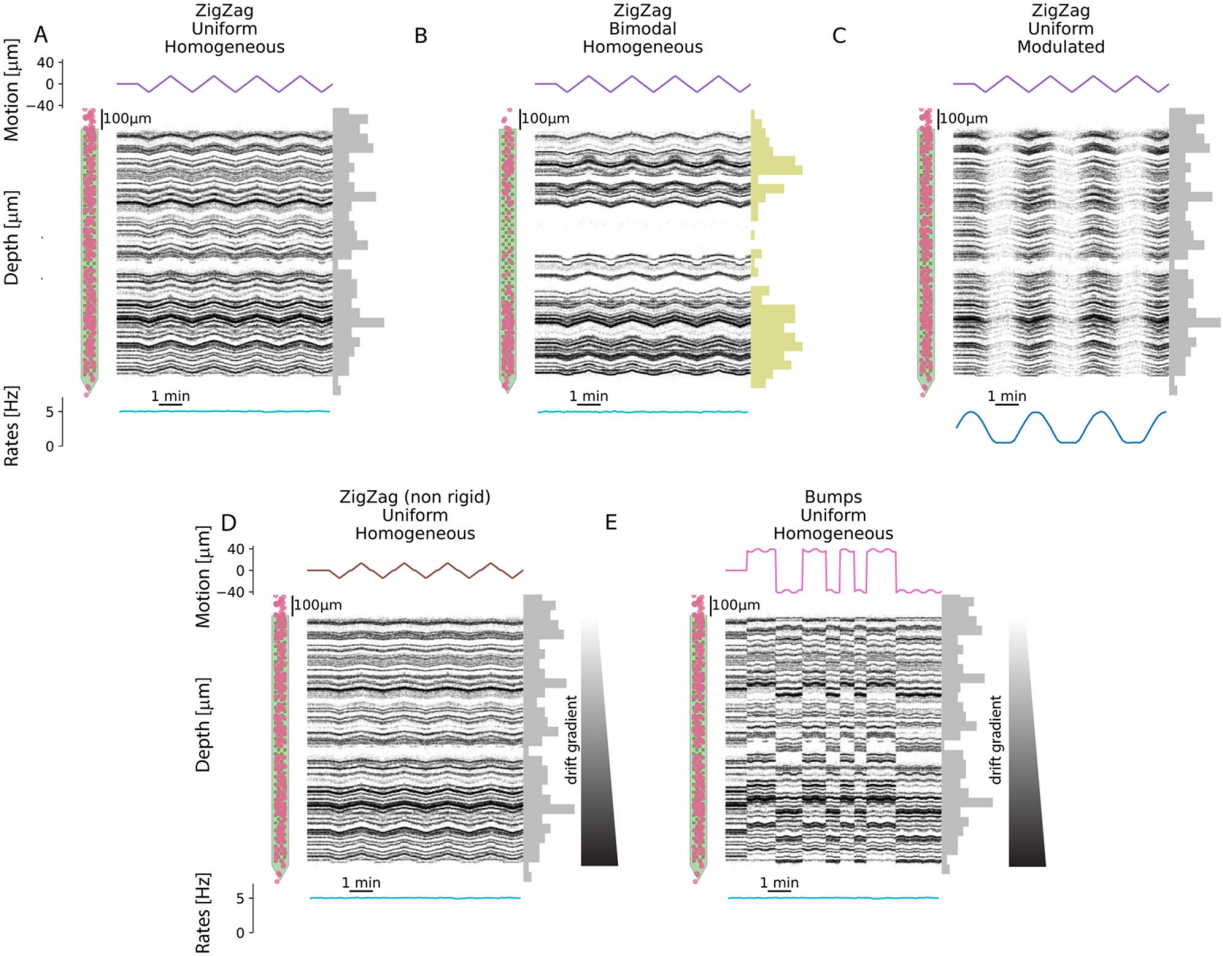
Examples of simulated drift recordings. For each panel, the top shows the layout of the probe with superimposed starting position of each cell (left), the positional raster plot with overlaid GT motion signals (center), and the depth distribution of the 256 neurons. The bottom part displays the firing rate modulation. Here we display 5 out of 12 recordings (the remaining 7 can be found as Extended data Figure on the github repo of the project). ***A***, Rigid ZigZag drift with uniform depth distribution and homogeneous firing rates. ***B***, Rigid ZigZag drift with bimodal depth distribution and homogeneous firing rates. ***C***, Non-rigid ZigZag with uniform depth distribution and modulated firing rates. ***D***, Non-rigid ZigZag with uniform depth distribution and homogeneous firing rates. ***E***, Bumps with uniform depth distribution and homogeneous firing rates.

The first obvious source of variability is the nature of the drift itself. Drifts can indeed be slow, rigid, or non-rigid as a function of the positions of the cells, but can also be abrupt and discontinuous, with jumps of few tens of microns (Steinmetz et al., 2021). We therefore generated three drift signals:

- *Zigzag (rigid):* For this drift mode, we used a rigid drift (all cells move homogeneously across depth) with a triangular slow oscillation, an amplitude of 30μm and a 30μm/min speed. Figure 2, *A* and *B*, shows sample raster maps using this drift mode.
- *Zigzag (non-rigid):* This mode is similar to Zigzag (rigid), but in this case, the drift is non-rigid, leading to a non-homogeneous movement of all the cells as a function of their depths. In this non-rigid case, the drift vector is modulated by a linear gradient, ranging from 0.4 ( 12μm maximum displacement) at the upper part of the probe to 1 ( 30μm maximum displacement) at the bottom. Figure 2, *C* and *D*, shows sample raster maps using this drift mode.
- *Bumps (non-rigid):* To reproduce the abrupt transient drifts that can often be observed in vivo (Durand et al., 2023), we also modeled fast, abrupt drifts that can happen irregularly during the recording. These abrupt events happened at random times (between 30 and 90 s) and moved all cells in a non-rigid manner, with minimal amplitudes between −20 and 20 μm reached at the top of the electrode and maximal ones between −40 and 40 μm at the bottom of the probe. The drift signals also experience an additional small 3 μm sinusoidal modulation with 40 Hz frequency. Figure 2*E* shows sample raster maps using this drift mode.

#### Depth distributions

A second feature that could affect motion estimation is the presence of probe regions with very low spiking activity. In fact, due to the paucity of localized spiking information, this might lead to a wrong estimation of motion. To account for this level of complexity, we simulated different depth distributions:

- *Uniform:* The depths of the selected neurons (256) were drawn from a uniform distribution, as in Figure 2*A* and *C*–*E*.
- *Bimodal:* The neuron depths follow a bimodal distribution with two peaks at the top and bottom of the probe and a central region with a lower density of cells, as in Figure 2*B*.

#### Firing rates

A third level of complexity is represented by non-stationary firing rates in the spiking activity. This scenario can be particularly challenging for motion inference methods that rely on an *average* template, such as the iterative template method. We therefore simulated two cases:

- *Homogeneous:* The activity of all neurons was modeled as homogeneous Poisson sources with 5 Hz mean firing rate, as in Figure 2*A*, *B*, *D*, and *E*.
- *Sine-modulated:* The spike trains were obtained following a non-homogeneous Poisson distribution modulated with a slow sine wave with a 3-min period, as in Figure 2*C*. In practice, to avoid too few spikes, the sinusoidal profile is clipped at 0.5 Hz.

We generated a total of 12 scenarios by combining the above-mentioned option (3 drift signals × 2 depth distributions × 2 firing rates). Five of these cases are shown in Figure 2, the rest can be found as a Extended data Figure available on the github repo of the project. In all cases, the drift started after 60 s, so that the first portion of the recording is drift-free. Owing to the reproducibility option of the MEArec simulator, which allows one to set random seeds to control all steps of the simulation, for each scenario we generated a *static* counterpart, with the exact same neurons, spiking activity, and noise profile, but only without drifts. We used these *static* recordings to benchmark the motion interpolation step.

### Evaluation

To evaluate the impact of the motion estimation step, we took advantage of the known GT motion signals at different depths. We computed the estimation error as the Euclidean distance between the GT motion and the one estimated by the different methods. Note that to compensate the offsets between estimated and GT motions, we aligned the medians of the GT and the estimated motions. From these error signals, we could then visualize both the evolution of the error over time and the error profile along the depth dimension (by averaging accordingly).

To benchmark motion interpolation, we used the GT motion signals to interpolate the traces and then utilized the GT spike timing to extract single spike waveforms from the motion-corrected and associated *static* recording. Assuming that the neuron *i* has fired *N* spikes with waveforms ${\vec{w}}_{i}^{1\;\ldots \;N}(t)$ (defined on a subset of their five best channels, i.e., where amplitudes are minimal), we calculated the dispersion around the mean template ${\vec{T}}_{i}(t)$ as the STD *σ*_*i*_ to measure of how “variable” these spikes are. Normalization is achieved by dividing *σ*_*i*_ by the root mean square (RMS) of the template ${\vec{T}}_{i}$. To compare the effect of different motion interpolation procedures, we further computed the ratio $\sigma_{i}^{\mathrm{interpolated}}/\sigma_{i}^{\mathrm{static}}$ for every neuron *i*. The rationale for such a metric is that spike sorting algorithms assume that waveforms are “sterotypical,” but drift is increasing the variability due to movements. Interpolation should therefore reduce this extra variability introduced by drifts.

Finally, and most importantly, we evaluated how drift correction affects spike sorting outcomes. To do so, we focused on the Kilosort 2.5 algorithm, which first introduced drift correction as a preprocessing step to spike sorting (Steinmetz et al., 2021). We ran all spike sorting jobs in the SpikeInterface framework (Buccino et al., 2020) on a 40-core Intel(R) Xeon(R) Silver 4210 CPU @ 2.20GHz, with 64GB of RAM and an 8GB Quadro RTX 4000 GPU. We used default parameters, but ran the preprocessing and motion correction steps in SpikeInterface instead of Kilosort 2.5. To match the Kilosort 2.5 processing, before motion correction, we applied a highpass filter (cut-off frequency at 150 Hz), a common median reference, and finally a local whitening step (Yger et al., 2018; Pachitariu et al., 2023; using, for each channel, the neighbor electrodes within 150 μm radius).

Knowing the GT spike timing, we could compute the *accuracy* of each GT unit *i* as

$$acc_{i}=\frac{TP_{ij}}{TP_{ij}-N_{i}-N_{j}}\;\;$$ (11)

where *j* is the sorted unit matched to the GT unit *i*, *N*_*i*_ and *N*_*j*_ are the number of spikes in the GT and matched sorted unit, respectively, and *TP*_*ij*_ is the number of *true positive* spikes, i.e., the spikes found both in the GT and sorted spike trains. From this accuracy metric, we further classified spike sorted units as:

- *well detected*: units with an accuracy greater than or equal to 80%.
- *overmerged*: units with an agreement above 20% with more than one GT unit.
- *redundant*: units with an agreement above 20% with a GT unit that are not the best matching unit. These units can be either oversplit or duplicated sorted units.
- *false positive*: sorted units with an agreement below 20%.
- *bad*: the sum of *overmerged*, *redundant*, and *false positive* units.

## Results

### Benchmarking motion estimation

**Figure 3.**
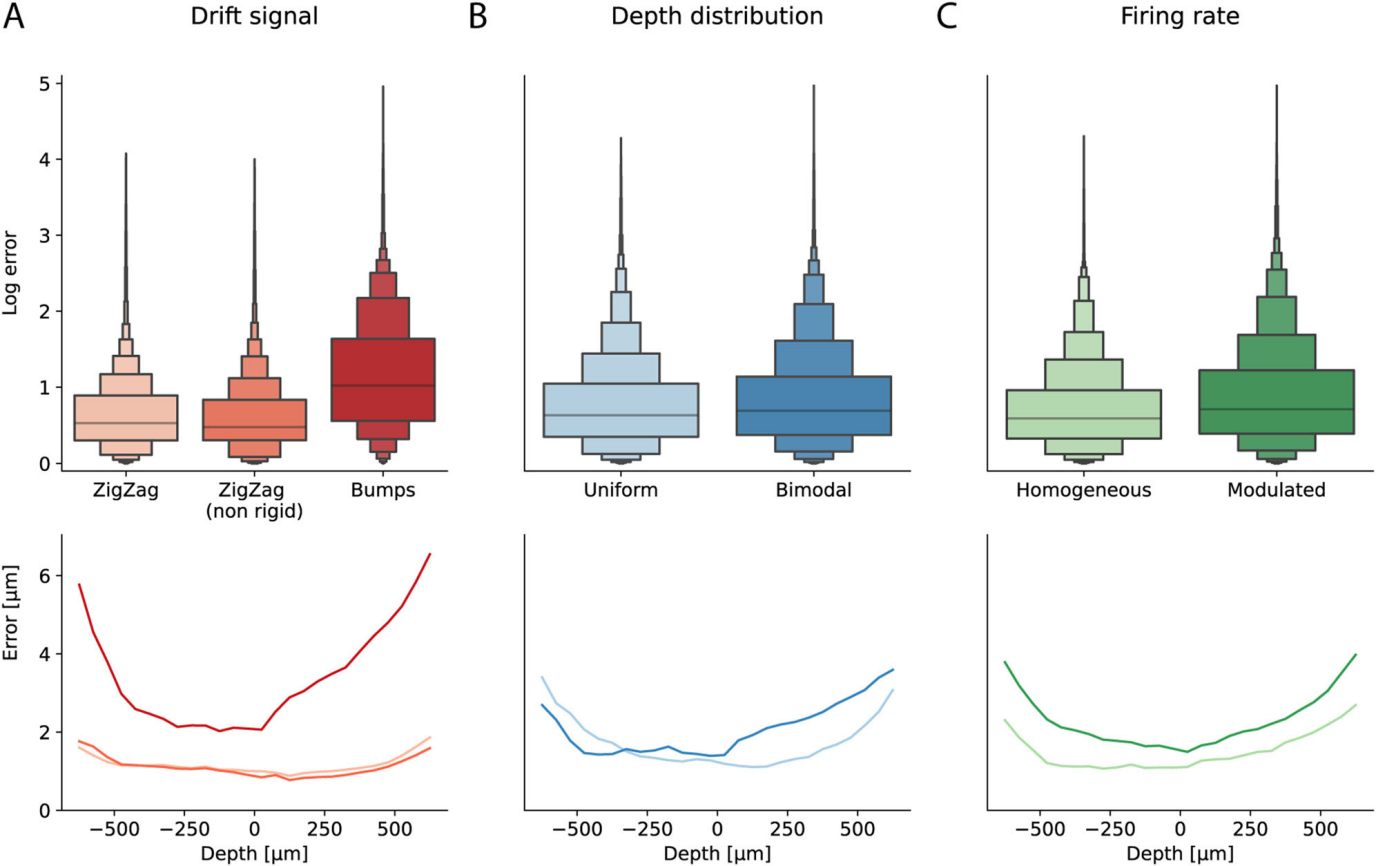
Motion estimation performance for different simulation modes. ***A***, Top: distribution of the log errors for all the errors pooled over all motion correction pipelines tested in this paper, as a function of the types of drift (ZigZag, ZigZag (non-rigid), or bumps). Bottom: averaged error over all methods, as a function of the probe depth and the nature of the drift. ***B***, Same as in *A*, but as a function of the type of the depth distribution (uniform and bimodal) of the neurons. ***C***, Same as in *A*, but as a function of the firing rate profiles.

To benchmark the performance of different motion estimation strategies, we generated several artificial recordings with 256 neurons and various drift cases (see Materials and Methods, Simulated datasets). Before diving into the fine details on the performance of different methods, we first looked at the global averaged errors over all algorithmic pipelines tested in this paper (see Methods); as a function of the drift features, we decided to explore while generating the artificial datasets. Since error samples are not independent (e.g., due to time and depth dependencies), we decided not to perform statistical test, but only qualitatively observe global trends. As in Figure 3*A*, we show the error distributions as a function of the drift signal type. Clearly, when drifts are discontinuous (bumps), all motion estimation algorithms show larger errors. Instead, there is no evident difference between the rigid and non-rigid ZigZag, which suggests that the methods that are used are properly able to handle non-rigid motion. Regarding the other drift features, a bimodal depth distribution results in slightly higher errors than a uniform one (Fig. 3*B*), and a modulate firing rate profile is more challenging than a homogeneous one (Fig. 3*C*). In all cases, another important general trend to highlight is that errors are larger at the borders of the probe (bottom panels). This is because channels at the borders have less spatial information to properly localize the peaks.

**Figure 4.**
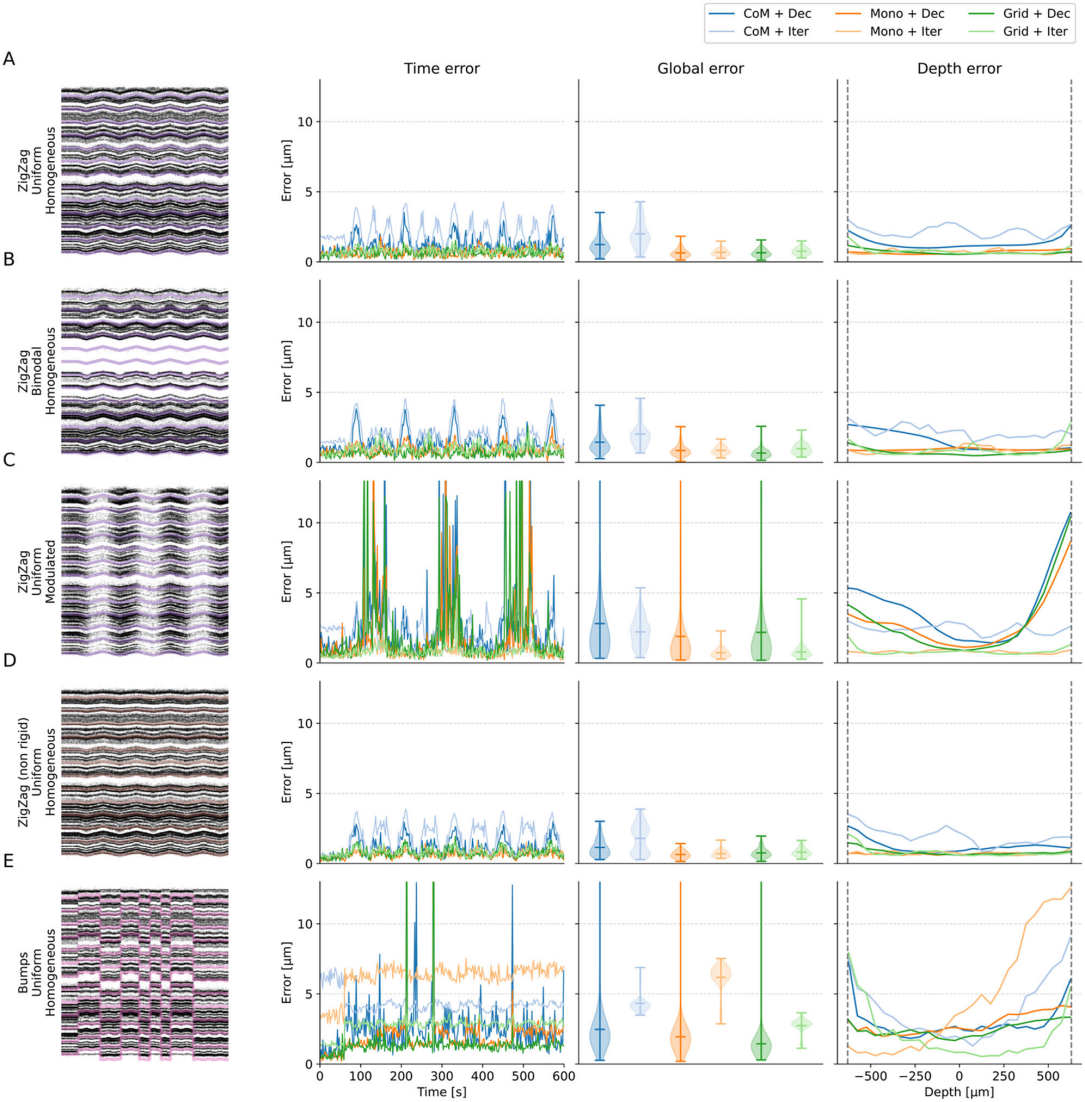
Performance in motion estimation for various drift scenarios. For various situations of the drifts with overlaid GT motion (left column), errors (from left to right) as a function of time (time error), averaged (global error), or as a function of the depths (depth error) and for various motion estimation pipelines. Here we display 5 out of 12 recordings (the remaining 7 can be found in the github repo of the project). ***A***, Zigzag (rigid), bimodal positions, homogeneous rates. ***B***, Zigzag (rigid), bimodal positions, homogeneous rates. ***C***, Zigzag (non-rigid), uniform positions, modulated rates. ***D***, Zigzag (non-rigid), uniform positions, homogeneous rates. ***E***, Bump (non-rigid), uniform positions, homogeneous rates.

**Figure 5.**
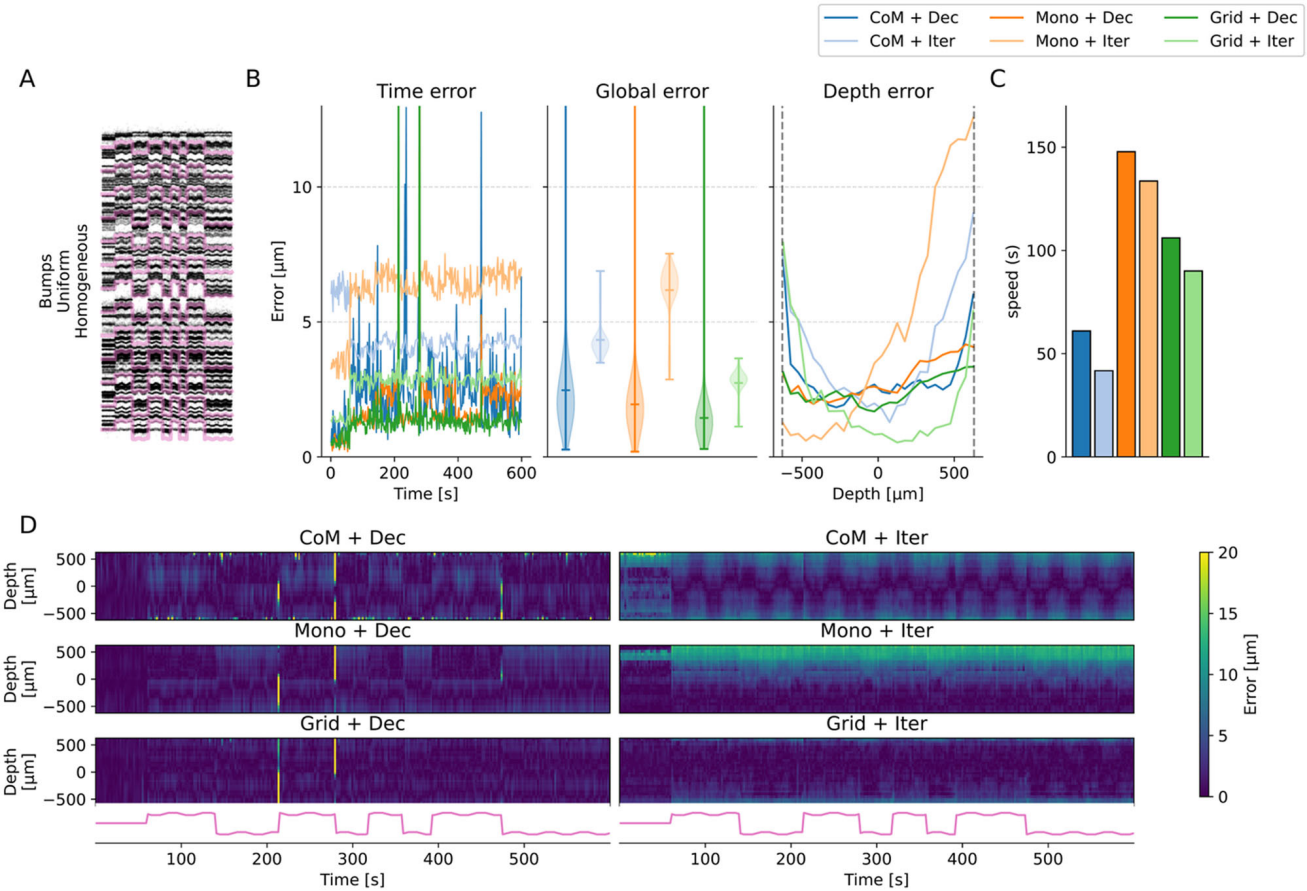
Motion estimation performance for the bumps (non-rigid) drift case. ***A***, Positional raster plot to show the movement of the cells, during the simulated drift. ***B***, The errors with respect to the GT motion vector as a function of the time (time error), averaged over time (global error), or as a function of the depth (depth error) for several motion estimation pipelines. ***C***, Run time of the different pipeline to estimate the motion (see legend below). ***D***, Errors, as a function of depth and time, for all the motion estimation methods considered. The time course of the averaged drift vector is shown at the bottom.

In Figure 4, we first show the results for a representative subset of five drift recordings (one per row). All the remaining cases can be found as a Extended data Figure available on the github repo of the project. The left column displays the raster maps with the overlaid drift signals at multiple depths, which give a qualitative view of the drift over the entire recording. The right part of the figure focuses on the errors of different *peak localization* + *motion inference* combinations: the left panels show the errors over time, the central panels the error distributions, and the right panels the errors over probe depth (left-bottom and right-top). The first thing that we should highlight is that motion estimation, irregardless of the method, is generally good. Average errors for relatively smooth drifts (*A*–*D*) are less than 5 μm, which is well below the inter-electrode distance. Even in the case of *Bumps* (*E* and *F*), where errors are more pronounced, some but not all methods can achieve average errors below 5 μm. Second, the errors tend to be larger at the borders of the probe. This is expected because we have less spatial information there, thus the motion inference is less accurate. Another general observation is related to the peak localization methods, used to estimate the activity profile: the CoM (*CoM*—blue color) method yields higher errors, and hence performs worse than all other estimation methods (but it is the fastest—see Fig. 5*C*). The monopolar triangulation (*Mono*—oranges) and the grid convolution (*Grid*—green) perform similarly, with the former achieving slightly lower error distributions. As for the motion inference step, the decentralized method (*Dec*—dark shades) generally produces lower errors than the iterative template (*Iter*—light shades) approach, except for the ZigZag/bimodal/modulated case (panel B). We should also note that the errors from the decentralized method, despite lower on average, show increased variance, due to some time bins being highly misestimated (see *spikes* in the second column of Fig. 4*B*, *E*, and *F*). During these particular time bins, the increased errors can dramatically affect the motion interpolation step and the overall spike sorting results.

To further investigate what is exactly happening during a complicated case, we decided to focus on the bumps/uniform/homogeneous case in Figure 5. As one can see in Figure 5*B*, the errors made by the decentralized algorithm (either via *CoM*—blue, *Mono*—orange, or *Grid*—green—localization) are lower, on average, compared to the ones made with the iterative template algorithm (see global errors). The method seems to be less sensitive to the depth (see Fig. 5*B*, depth error), with lower errors on average. The computational cost of the motion estimation part is slightly higher (see Fig. 5*C*), but most of the computational cost is due to the peak localization methods (the monopolar approximation is more accurate, but also slower as the results of an optimization problem). This is a point not explored here, since we are computing the position for all peaks, but only in a 10-min-long recording. For longer recordings, to limit the computational burden, sub-sampling the peaks prior to localization might be necessary (in the current implementation). Figure 5*D* shows the errors as a function of both depth and time, for all motion estimation options. Large transient errors (yellow stripes) can be observed for the decentralized method (and also in one case for the iterative template method) and do not seem to be correlated with depths. Such large errors, although transient, can have a very negative impact at the spike sorting level, because during these time bins, spikes are unlikely to be correctly recovered.

### Benchmarking motion interpolation

After quantifying the performance of motion estimation, we evaluated and compared different motion interpolation methods: snapping (Snap), inverse distance weighting (IDW), and kriging (Krig) (see Materials and Methods, Motion interpolation for details). Note that kriging is the method implemented in Kilosort (Pachitariu et al., 2023).

**Figure 6.**
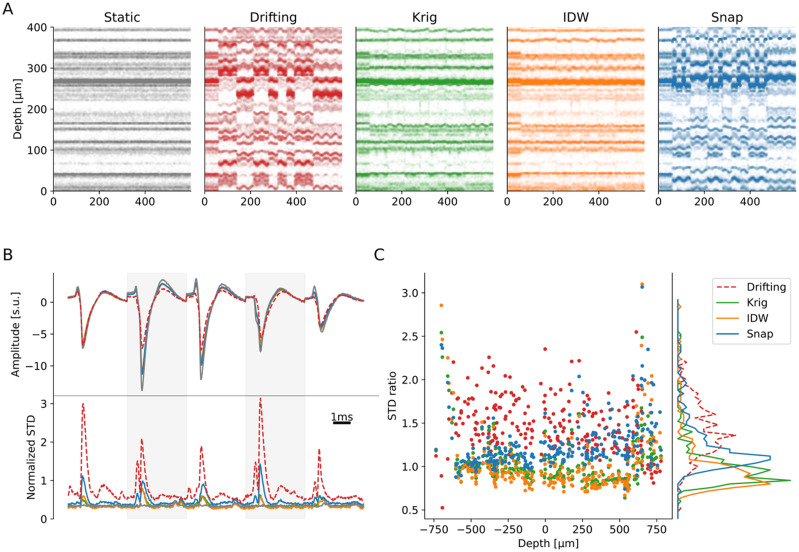
Quantification of the interpolation methods. ***A***, Positional raster plots with a bumps (non-rigid) drift (uniform/homogeneous) for the static (gray), drifting (red), and interpolated traces *via* Kriging (green), IDW (orange), or Snap (blue), using the GT motion vector. Note that the probe is cropped here for visibility purposes. ***B***, Top: flattened template (on a subset of channels) for a single neuron in all the conditions (static, drifting, and the three interpolation methods), in units of the median absolute deviation (mad) of the noise on the considered channels. Bottom: STD over time, normalized by the template RMS, of all the individual spikes for this particular neuron, in the same conditions. ***C***, Ratios of waveform STD compared to the static case for all the cells in the recording, in all conditions, and as a function of the depth of the neurons. Right panel displays the overall STD ratio distributions. In this case, an STD ratio above 1 means an increase in waveforms dispersion with respect to the static case (and a decrease for ratios below 1).

As a representative example, we focused on one recording (bumps/uniform/homogeneous) for this section. Figure 6*A* shows a portion of the raster maps (from 0 to 400 μm depth) for the *static* (gray) and drifting (red) recordings, and the interpolated recordings with kriging (green), IDW (orange), and snapping (blue). The raster maps are estimated from the interpolated traces after applying filtering, common median referencing, and z-scoring and using monopolar triangulation to estimate peak depths. To isolate the effects of interpolation, we used here the GT motion vector as input for the various interpolation methods. Already from the raster maps, one can see that the kriging and IDW procedures seem to compensate well for the added motion, but in both cases some *wiggles* can be observed, indicating imperfections in the interpolation. In the ideal case, after interpolation, one would expect straight bands similar to the *static* case. The snapping method, instead, is not capable to compensate for such large drifts, also due to the relatively low spatial density of the Neuropixels 1.0 configuration (with electrode distances of ∼20μm). Higher electrode densities might improve the results of this method.

To have a better insight on how interpolation affects spike waveforms, in Figure 6*B* we show the average template of one unit (top) and the temporal variance around the template (bottom) on the five channels with the largest amplitude. It is important to look at waveform variability since the main assumption of all spike sorting algorithms is that waveforms from a given neuron are reliable and stationary. A deviation from this assumption will therefore likely translate to worse spike sorting performances. For all interpolation methods, the interpolated template is smaller than the static counterpart (gray) and larger than the drifting recording (dashed red). This is probably due to the inevitable spatial smoothing that results from averaging the signal from different channels (for kriging and IDW). The temporal evolution of the STD also shows an increase, specifically in correspondence of the template peaks. Nevertheless, the STD is largely reduced with respect to the drifting case, indicating that motion interpolation *should* improve spike sorting results.

In Figure 6*C*, we show, for each neuron, the ratio of the waveform STD with respect to the static waveforms, depending on the neuron depth (left) and the distribution of these ratios (right) for different cases (red—drifting, green—kriging, orange—IDW, blue—snapping). We remind that this view is an idealized situation in the drift correction pipeline, because here the exact GT motion vector is provided to interpolate the traces. As expected, the waveform variability is systematically increased for all cells in the drifting case compared to static. For IDW and kriging, the overall ratio not only is reduced, but it is even less than one on average, probably due to the spatial smoothing resulting from the interpolation procedures, which reduces the additive uncorrelated noise. Since the test recording has non-rigid drifts, we can also observe a slight correlation with depth, with smaller variabilities as the depth gets positive (and the drift amount reduces). A strong border effect at the borders of the probe is also visible, with STD ratios increasing regardless of the methods. This is also expected, since all motion interpolation procedures, close to the probe borders, can only rely on partial information to interpolate the traces.

### Global impact at the spike sorting level

After evaluating the performance at the motion estimation and motion interpolation stage, we now investigate how a complete drift correction pipeline affects spike sorting results. From the previous section, we showed that IDW and kriging are the best interpolation options. For this analysis, we used kriging since it is also used by Kilosort 2.5 and it therefore allowed us to perform a direct comparison.

We ran spike sorting using Kilosort 2.5 on three different datasets—ZigZag, ZigZag (non-rigid), and bumps—all with uniform depth distributions and homogeneous firing rates (Fig. 2*A*, *C*, and *E*). For each recording, we ran five spike sorting options, and note that the cell distributions and spiking activity for all three recordings are the same, resulting in the exact same *static* recordings:

- *Static—no interpolation:* spike sorting on the *static* recording, turning off motion correction in Kilosort 2.5
- *Static—using KS2.5:* spike sorting on the *static* recording, turning on motion correction in Kilosort 2.5
- *Using GT:* spike sorting on the drifting recording, using the GT motion signals and kriging for motion interpolation in SpikeInterface
- *Using Mono + Dec:* spike sorting on the drifting recording, using the estimated motion with monopolar triangulation + decentralized inference for motion estimation and kriging for motion interpolation in SpikeInterface
- *Using KS2.5:* spike sorting on the drifting recording, letting Kilosort 2.5 preprocess the recording and correct for drifts (equivalent to grid localization + iterative template inference for motion estimation and kriging for motion interpolation).

**Figure 7.**
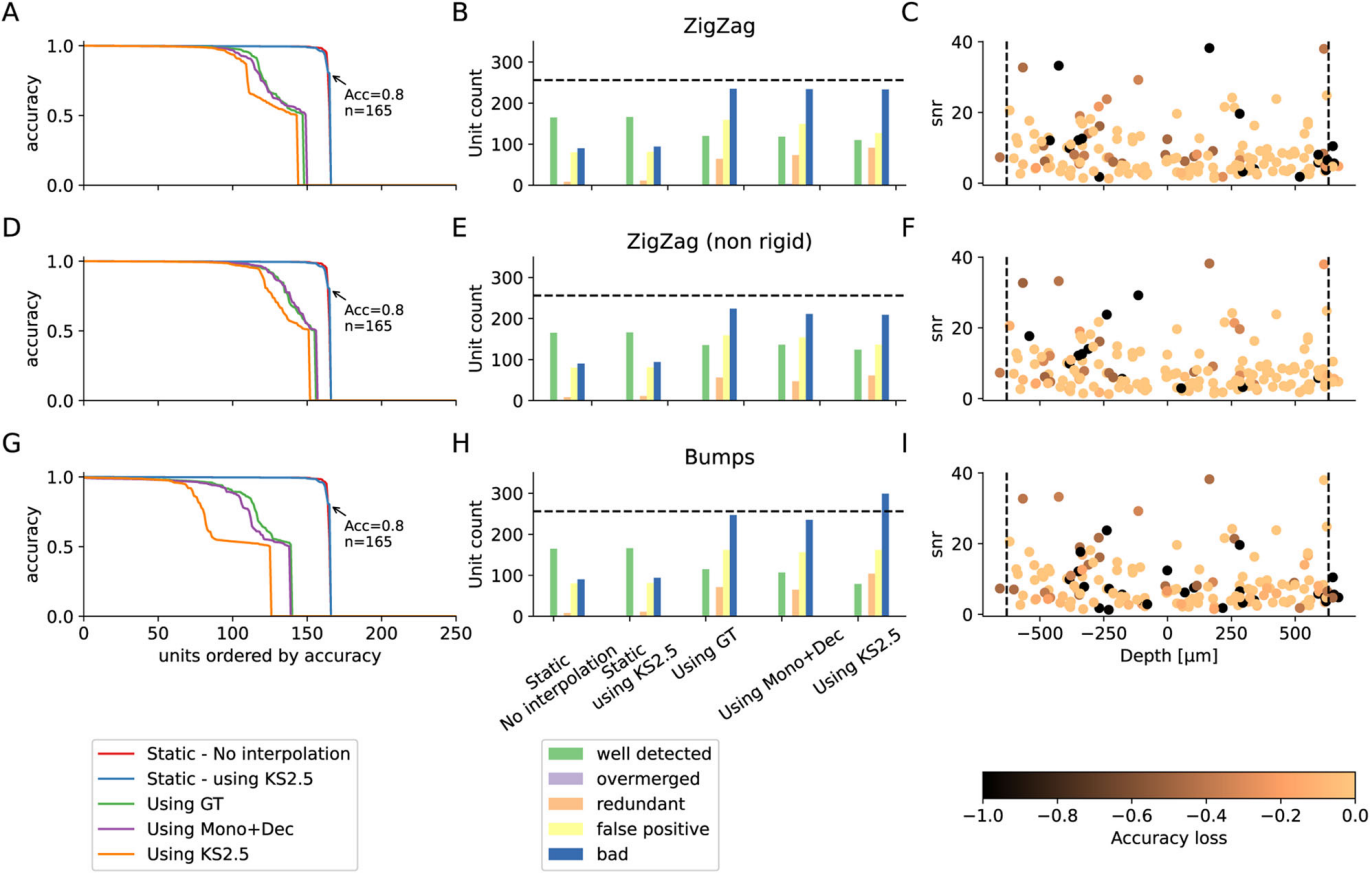
Quantification of the interpolation on sorting accuracy. ***A***, ***D***, ***G***, Accuracy of all units (sorted by accuracy) in the simulated recordings when launching Kilosort 2.5 in various conditions for the Zigzag rigid case (***A***), the Zigzag (non-rigid case) (***D***), or the bumps (non-rigid) case (***G***). All recordings have uniform distributions and homogeneous firing rates. ***B***, ***E***, ***H***, Number of well detected/overmerged/redundant/false positive and bad units found by Kilosort , with various drift correction methods, for the recordings explained in *A, D, G*. ***C***, ***F***, ***I***, The loss in accuracy for the well-detected neurons (accuracy higher than 0.8—*N* = 164) between the static case and the one using Mono + Dec estimation, as a function of the depth of the neurons (*x* axis) and the SNR (*y* axis).

Figure 7, *A*, *D*, and *G*, shows the accuracy of the spike sorting for all GT units (sorted by accuracy). For all test cases, we observe a lower performance on the drift-corrected recordings with respect to the static ones (red lines). This drop is particularly dramatic in the complex case of Bumps (Fig. 7*G*). Nevertheless, running motion correction using the Mono + Dec estimation in SpikeInterface (green lines) produces better results than using the Kilosort 2.5 correction procedure (purple lines) and it yields similar performance to using the GT motion signals for interpolation (blue lines). In some cases, the accuracy using the estimated motion is even slightly better than the one using GT signals. This could be explained by the fact that the estimated motion is fully data-driven, while the effect of the GT motion on the traces can also depend on the neuron location, morphology, and relative orientation with respect to the electrodes. Note that even in all cases, the accuracy loss is rather large, indicating that the interpolation step is degrading spike sorting outputs. It is however interesting to note that the motion correction feature of Kilosort 2.5 is not harming the results when no drift is present. This is because when Kilosort does not detect any drift, no interpolation of the traces is performed and thus the extracellular traces are not modified, leading to very similar results with respect to static recordings (the slight differences are due to run-to-run variability of Kilosort itself).

When we look at the classification of sorted units (Fig. 7*B*, *E*, and *H*), the number of well-detected units is 165 for the static recording, but it drops to 118 for Mono + Dec and 110 for Kilosort on the ZigZag rigid recording; 136 for Mono + Dec and 124 for Kilosort on the ZigZag non-rigid recording; and 107 for Mono + Dec and 79 for Kilosort on the Bumps recording. In addition, a much larger number of bad units is observed after drift correction, resulting from both false positive and redundant units. However, correcting with SpikeInterface also results in a reduced number of bad units with respect to Kilosort : for example, for the Bumps example, Mono + Dec finds 238 bad units and Kilosort finds 302.

In Figure 7*C*, *F*, and *I*, we display the drop in accuracy for the well-detected units in the static recording (*N* = 165) between the static and the *estimated Mono + Dec* cases depending on the unit depths (*x* axis) and signal-to-noise ratio (SNR; *y* axis). While a clear trend is not apparent, some units with large SNR seem to exhibit a large drop in accuracy. These drops in accuracy could partially be due to oversplits due to residual drifts, which would also explain the large number of redundant units. In order to assess this, we performed a merging procedure of the sorted results using the GT information. In brief, for each GT unit, all the sorted units with an accuracy greater than or equal to 20% were merged into a single unit. Even after this merging step, there is still an observable drop in accuracy after interpolation compared to static (see Extended data Fig. 3*A*, *D*, and *G*, available on the github repo of the project). The number of well-detected units is increased, redundant units are almost gone, and the accuracy drops of high-SNR units are mostly recovered. However, we want to highlight that this precise merging procedure is only possible for GT data, and other strategies will need to be employed for experimental data, such as manual merging or alternative data-driven automated methods (Llobet et al., 2022).

### Drift correction with SpikeInterface

In the previous sections, we benchmarked the different steps involved in drift correction and their global impact on spike sorting outcomes. As mentioned in Section “A modular implementation of drift correction,” we implemented all the methods in SpikeInterface with a modular architecture. For example, here is a code snippet that shows how to estimate motion signals from a Neuropixels recording:

import spikeinterface as si

import spikeinterface.extractors as se

# modular implementation

from spikeinterface.sortingcomponents.peak_detection import detect_peaks

from spikeinterface.sortingcomponents.peak_localization import detect_peaks

from spikeinterface.sortingcomponents.motion_estimation import estimate_motion

from spikeinterface.sortingcomponents.motion_interpolation import interpolate_motion

# Load Neuropixels recording

recording = se.read_spikeglx(spikeglx_folder)

# Step 1: peak detection

peaks = detect_peaks(recording,

method='locally_exclusive'

)

# Step 2: peak localization

peak_locations = localize_peaks(recording,

peaks,

method='monopolar_triangulation'

)

# Step 3: motion estimation

estimated_motion, temporal_bins, spatial_bins = estimate_motion(recording,

peaks,

peak_locations,

method='decentralized'

)

# Step 4: motion interpolation

recording_corrected = interpolate_motion(recording,

estimated_motion,

temporal_bins,

spatial_bins,

method='kriging',

border_mode='remove_channels'

)

Note that the border_mode=’remove_channels’ removes the channels at the border of the probe based on the maximum estimated motion and should reduce the inaccuracies observed at the borders of the probe.

## Discussion

In this work, we have presented a comparison of state-of-the-art methods to correct for motion in extracellular electrophysiology recordings. We generated a wide variety of simulated benchmark datasets using a Neuropixels 1.0 probe design with varying levels of complexity, using Zigzag and/or *bumping* drift signals, rigid or non-rigid motion, and uniform or non-uniform depth distributions and firing rates. Knowing the GT motion and spiking activity, we were able to finely evaluate the two different steps involved in drift correction, i.e., motion estimation and motion interpolation.

For motion estimation, we showed that the combination of monopolar triangulation (for peak localization) and decentralized registration generally outperforms all other options on the test datasets. Nevertheless, in its current implementation, localizing peaks with monopolar triangulation is more computationally expensive than other strategies (center of mass and grid interpolation), and the decentralized motion estimation method has a tendency to provide short but high spurious errors during some time bins. It is also important to notice that the accuracy in estimating motion signals is lower close to the borders of the probe, owing to the partial view of drifting activity with neurons that might move in and out of the *field of view* and thus be harder to track.

To evaluate motion interpolation, we compared the motion-corrected traces to a matching *static* simulation without added drift. We found that the *kriging* method (same interpolation method as 2.5; Pachitariu et al., 2023) achieves an equally good performance in reconstructing the static traces compared to IDW scheme. Similarly to the drift estimation, the activity at the borders of the probe cannot be fully recovered/interpolated, because of missing information. Therefore, since both motion estimation and interpolation perform poorly at the borders of the probe, it would be advisable, for recordings with apparent drift, to discard portions of the probe close to the borders from downstream analysis. The design of metrics and/or quantitative criteria to perform such a slicing of the probes should be the subject of further studies and have already been implemented in SpikeInterface.

Finally, we evaluate the overall impact of the drift correction on spike sorting results. We found that applying the best motion estimation strategy (monopolar + decentralized registration) outperforms the Kilosort 2.5 implementation (grid interpolation + iterative template registration) and yields the same accuracy as using GT motion signals for the motion interpolation step. Importantly, the reader should note that even applying the *best* drift correction dramatically reduces spike sorting accuracy with respect to a static recording. In other words, even using the best correction strategy will not recover the same spike sorting accuracy as with a static recording. Electrophysiologists should therefore pay additional care in trying to minimize any major source of drifts, for example, by stabilizing the rig, lowering insertion speeds (Fiáth et al., 2019), and partially retracting the probe after insertion (Durand et al., 2023). On the algorithmic side, re-interpolating the input traces to correct for drifts prior to spike sorting might not be the favorable solution moving forward, since not only the waveforms are distorted but also the noise levels. Given the high accuracy of the motion estimation step, an alternative approach could therefore be to use the motion signals *within* the spike sorting pipeline, for example, by correcting waveform features before clustering or by using evolving templates over time in template matching (Boussard et al., 2023).

In this work, we used only simulated datasets on Neuropixels 1.0-like probe designs as a benchmark. While clearly artificial data cannot fully substitute experimental ones, they provide a very controlled and customizable framework to test specific aspects of recordings, including drifts. A quantitative evaluation of drift correction methods on real data would in fact be very challenging due to the lack of GT. Even in the case of mechanical manipulation of the probe to inject drift (Steinmetz, 2021), the relative movement of different layers of tissue with respect to the rigid probe movement would be hard to control. Furthermore, while here we focus on Neuropixels 1.0 probes, commercialized in 2019 and already widely used in neuroscience research, further benchmarks following a similar approach could target different probe layouts, such as Neuropixels 2.0 (Steinmetz et al., 2021), Neuropixels Ultra (a prototype version with closely packed electrodes at 5 μm pitch Andrew M Shelton et al., 2023), or other HD-MEA probe designs, such as the SiNAPS probe (Angotzi et al., 2019). Different probe geometries and electrode densities could in fact affect motion correction in non-trivial ways. The Neuropixels 2.0 layout was designed with drift correction in mind and is believed to improve the performance of motion correction with respect to the staggered layout of Neuropixels 1.0 (Steinmetz, 2021). For such configurable probes, users should keep in mind that choosing lower density configurations might badly affect the motion correction step. In this light, the presented simulation-based benchmark could be used in the in silico test and design of novel, more drift-resistant, probe layouts and geometries. It is also worthwhile noting that behavioral brain states might also have a direct impact on motion correction pipelines. However, as for real experimental data, such states can be hard to model and might depend a lot on the animals and experimental conditions. Finally, in this work we did not explore the relatively large parameter space associated to the different methods that we used, but we rather chose default parameters suggested by the original implementations. Future improvements could target fine-tuning the parameters to further improve the performance of motion correction methods.

All motion estimation methods evaluated in this analysis use the detected peaks to construct activity histograms utilized to reconstruct drift signals. While these approaches are appropriate for recordings in rodents, they may fall short when dealing with recordings experiencing drifts at faster scales, such as the heart-beat and breathing modulations observed in recent recordings from humans (Paulk et al., 2022; similar drifts could be expected from non-human primates; Trautmann et al., 2023). For drift at such fast timescales, local field potential signals can be used instead of peak location estimates to build activity histograms (Paulk et al., 2022; Windolf et al., 2023).

Finally, all the methods and drift correction options evaluated in this work are readily available to the electrophysiology community within the SpikeInterface package and can be immediately deployed with a few lines of code prior to any spike sorting process, as shown in Section “Drift correction with SpikeInterface.”

## Synthesis

Reviewing Editor: Arvind Kumar, KTH Royal Institute of Technology Decisions are customarily a result of the Reviewing Editor and the peer reviewers coming together and discussing their recommendations until a consensus is reached. When revisions are invited, a fact-based synthesis statement explaining their decision and outlining what is needed to prepare a revision will be listed below. The following reviewer(s) agreed to reveal their identity: Michael Denker, Fabian Kloosterman.

Synthesis

Authors have addressed an important problem related to spike sorting. The reviewers think that the results are novel and can have the potential to be useful to the community. However, they raised two major concerns

The motivation and justification for the for the choice of simulations is not clear and often these choices lack concrete links to the neurobiology

The methods are not properly described. As you will see in the detailed comments, either the methods are obscure or incomplete in many places. Overall authors should rework the presentation of their work to make it easier for the reader. The two reviewers have made numerous suggestions for this.

The detailed comments of the reviewers are appended below. We look forward to reading your revision and reply to the reviewers' comments.

Reviewer comments #1

The content of the manuscript is novel and seems in large convincing. Indeed, information to assist experimentalists in setting up their spike sorting analysis pre-and post-processing stages is valuable. While I do believe this study adds to this accumulating evidence, the limitation of the study to consider data in a few selected example simulation outcomes without making a more concrete link to biology may decrease the enthusiasm a little. For this reason, below I suggest to include in the manuscript also the pipeline as a re-usable tool for researchers to adapt the example scenarios to their own needs in determining the optimal algorithms.

In addition, I found the presentation of the material requires additional work in terms of the writing style, description of methodology (which is somewhat confusing and unclear due to inconsistencies and missing information) and in figure design (which are largely too small and not well designed). Some examples of this, alongside additional issues encountered, are listed below.

Major issues:

- The description of methods, in general, is difficult to understand, partly missing information and partly being not fully consistent.

- COG:

- How is a waveform defined?

- How is it detected that a spike (meaning putative spike, given that it could be noise?) is present on multiple channels?

- What is ptp_i(c)? Which peaks are used in the calculation? Peak-to-trough?

- Grid:

- Am I correct that the described procedure is performed channel-by-channel, such that templates are created for one particular channel and then all channels are convolved with these templates? I did not fully understand the logic of the algorithm.

- What is the (typical) range of parameter k, 10 or 10000?

- The use of w_i conflicts with its use in COG. How is w_i calculated as representation of the spike itself? The average over w_i over channels all channels? Please clarify, I think the term "spike" is used partly as spike of a neuron, partly as synonym for threshold crossing.

- Do f_n really constitute a basis?

- Mono:

- In Eq. 5, I assume k is k_i

- Is z clamped to zero for the Neuropixel probe?

- Motion inference: In general, I found the short descriptions hard to follow. While I understand that a complete run-down of these arguments is out-of-scope for this manuscript, I would suggest to improve clarity and precision of the write up. For example, is spike depth the same as waveform peak-to-trough as in ptp? What amplitude is taken? What is meant by bin, and how is the correlation calculated -- the correlation of all bins in the shifted versus the target? How is the final drift vector shown as red line in Fig. 1C produced from the estimates?

- Similarly, the motion correction section could be reworked for more clarity to improve comprehension.

- Line 188: What was the motivation to use layer 5 neuron models throughout the depths? Would a more diverse template library affect results? Also, it would be interesting to see the choice of templates and the corresponding effects to judge the difficulty of the problem.

- Line 192: I am not sure I understand what is meant by the drift templates.

- Line 202ff: How were the drift amplitudes chosen for the various scenarios? Is this not a crucial parameter that may affect the benchmark, such that the drift amplitude should be varied as a parameter?

- Line 244: How is the dispersion dealing with waveforms of one emitted spike visible on different channels ("a subset of channels")?

- Line 291ff: Figure 3 displays strong oscillations in the time error, especially for the COG method. How is this explainable? It seems that without those oscillations, COG would be quite on par w.r.t. errors.

- Figure 4: Why is there such a large jump in error at the start of the time-error plot, especially for orange?

- Line 310: Is speed really an issue here? It seems that the algorithms differ at most by a factor of 4 -- and parallelization could probably help as many operations are suitable for embarrassingly trivial parallelization. In addition, is this a fair comparison in terms of having optimal implementations for all methods -- maybe it's just non-optimal in the way someone programmed it?

- Given that the manuscript can handle only a selected set of example scenarios, optimally the authors should provide their analysis pipeline in such a way that readers could -- with acceptable effort -- re-use it to create simulations adapted to their own scenario. I do understand this is not easy given the difficulties to package and integrate software such as Kilosort, but I would urge the authors to give it a best effort under https://github.com/to_be_updated_after_review and describe very briefly the available pipeline in the manuscript.

Minor issues:

- In general, the text reads in parts somewhat colloquial and could be polished more throughout. Examples of this are,

- A redundant "as one can see" in the caption of Figure 1

- "A round of peak detection is performed" in line 73

- Unnecessary suggestive "important" in line 308

- Line 79: Instead of inverse motion signal, I assume, negative or inverted is meant.

- Figure 1

- The figure is strange in the proportions of its constituent elements, especially the graphs can hardly be read even on maximum zoom levels.

- Panel A: please explain the panel in the caption, I was unable to interpret the figure. What are the template rows and columns? Does "Drifting positions" mean "positions after drifting", or does the figure show the dynamics during a drift?

- Panel B: The meaning of the blue boxes did not become clear from the description.

- Panel C: Why are activity profile estimation and motion inference lumped into a single (redish) box labeled motion estimation (which reads to me synonym to motion inference)? The same terminology is used in the text. I found this ambiguity a little confusing, but maybe those are established terms/hierarchies.

- Figure 2

- The individual drift traces are very hard to read in this figure. The authors should try to improve their presentation of results in terms of readability, also in later Figures.

- The rate profiles are not sinusoidal, but seem cropped towards zero -- likely due to the low likelihood to have a spike here. I would suggest to superimpose the the theoretical rate profile used for generation.

- Figure 3

- Parts of the figure caption seem to be in the main text (line 280-). I would suggest to improve separation between text and caption to improve readibility

- The overlay graphs are very difficult to read, especially for the time error.

- It would help to introduce time error, global error and depth error in the Methods.

- Figure 4

- Beside problems similar to Figure 3, the bar graph legends are nearly impossible to decipher without a good zoom functionality of the pdf reader

- Figure 4C could be improved by using more tightly spaced patterns or different shades of the respective bar colours instead. Here, the legend covers a ticklabel on the y-axis.

- Figure 6 uses similar colours for the categorical distinction in panels B, E, H and to map to the continuous accuracy values in panels C, F, I. Changing one of them could help to avoid potential misunderstandings. The legend for panels B, E, H seems to use internal variable names instead of more descriptive labels.

- Line 303: Why is the COG (blue) excluded? Here also, iter and dec show the same trend

- Line 314: I would like to better understand these yellow stripes and what is the cause here.

- Separating figure panels with dashes seems confusing since it could also refer to a range of panels.

- The spacing between numbers and units in the text is inconsistent.

- The dimensionality of some vectors was not clear to me from the text, e.g. $T_i(t)$ on line 248. Waveform length times number of channels? In general, terms like spike, waveform, template, etc should be well defined to reduce confusion.

Incomplete list of typos etc:

10: motion correction algorithm --> a motion correction algorithm

18: population --> populations

18: via is slanted

20: comparisons --> comparison

69: as outlined as follows --> as follows

74: as function of --> as a function of

75: a time windows --> a time window

86: Recent work --> Recent works

95: allows us first to --> enables us to

105: an histogram --> a histogram

106: as function of --> as a function of

107: negative thresholds -> negative threshold

136: with cost function --> with the cost function

168: the the --> the

181: words missing? "as exponentiated negative the distance matrix"

183: $1e^{-3}$ --> $10^{-3}$ (?)

192-193: on along --> along

288: the errors tends --> the errors tend

292: misplaced comma

311: 4D panel --> panel 4D

397: Figure --> In Figure

Reviewer comments #2

The authors present a modular open-source software pipeline to correct for drift in extracellular recordings. Drift - the relative movement of neuronal sources to the recording electrode - presents a challenge for long-duration extracellular recordings of spiking activity as it interferes with the task of assigning spikes to their neuronal sources (i.e., spike sorting). High-density neural probes provide an opportunity to track and correct for any drift by virtue of the large number of electrodes and hence the large number of cells that can be monitored. While the authors do not implement a new drift correction algorithm, they have collected a number of state-of-the art algorithms into a common framework (SpikeInterface) that facilitates comparison between algorithms. In addition, the authors decomposed existing drift correction algorithms into a set of three general steps that can be combined in different ways and which facilitates analysis of the contribution of each step to the overall drift correction performance. Finally, the authors performed benchmarking of the implemented drift correction algorithms on simulated data sets with varying types of drift. Overall, the work attempts to tackle an important issue in the field and the benchmarking provides an initial assessment of how well the state-of-the-art drift correction approaches perform. The results have the potential to be very valuable to the field.

My main concern is with the set of simulated signals that the authors have created and the rationale for combining variations in spatial-temporal drift and spiking activity dynamics. The 12 simulated signals are both redundant and unnecessarily complex for what the authors are trying to show and an opportunity is missed to illustrate how sensitive the tested drift correction approaches are to each of the aspects of drift and spiking activity dynamics. It would have been more informative if the impact of various aspects of drift and spiking activity on drift correct were assessed separately (unless there is a need to look at specific interactions).

Relatedly, the rationale for why particular drift/spike activity variants are picked and the potential impact on the drift correction performance (given knowledge about the general drift correction approach) is not clearly explained. For example, for the drift signals, it is only stated that "Different types of drift might challenge the drift correction approaches in different ways." I assume that the choice for the zigzag and bump style drift signals is (in part) driven by the question how the drift correction algorithms handle slow or fast drift speeds and the methods/results could be presented as such.

Further, it would be helpful for the authors to improve the description of the assumptions of the drift correct methods and provide more insight into the factors that impact drift correction performance. If I understand correctly, all presented drift correction procedures rely on a stable and non-uniform spatial distribution of spiking rates in the brain regions that are covered by the probe, either at the global scale (for rigid drift) or local scale (non-rigid drift). Any factors that violate these assumptions or that make it more difficult to obtain a good estimate of the spike rate distribution will likely hamper the drift correction performance. For example, a lower spiking rate or shorter time bins will reduce signal-to-noise ratio and increase variability in the estimate of the activity profile.

Other Major Comments

1. The test signals are all sampled in a single contiguous block of densely packed electrodes. With newer multi-shank probes (e.g. NP2.0) that have many more electrodes than available read-out channels, experimenters often need to distribute their read-out channels across the shanks in smaller non-contiguous blocks or reduce the density at which signals are sampled. It would be useful for the authors to comment on the impact such customized channel maps may have on the drift correction performance.

2. The authors simulated the spike trains of 250 neurons with locations randomly drawn from a uniform or bimodal distribution and either constant or sine-modulated firing rate.

2a. First a note that, as far as I understand, a uniform activity profile is not desirable for drift correction. The authors probably got away with using a uniform location distribution because the number of neurons per mm along the shank is sufficiently sparse, which by chance resulted in a non-uniform activity profile.

2b. As rationale for using uniform and bimodal location distributions for neuron location, the authors state that the distribution of neurons along the probe "might result in portions of the probe experiencing lower spiking activity". This requires further explanation, also because it appears to conflate the issues of spiking rate and spatial distribution of activity. For global (using all electrodes) correction of rigid drift, low spiking activity in portions of the probe contributes to a non-uniform activity profile and may thus be beneficial. On the other hand, for algorithms that use smaller portions of the probe to perform local correction of non-rigid drift, low spiking activity in those portions will pose a challenge.

2c. As rationale for using constant and sine-modulated spike rates, the authors state that "drift correction methods may assume stationary firing rates". Again, more explanation is needed on why drift correction method assume stationary firing rates. Additionally, the sine-modulated test signal may test the impact of low spiking rates rather than non-stationarity.

2d. In the results, no actual comparison is performed for the two neuron location distributions or for the two firing rate scenarios.

3. One challenge for drift correction procedures are behavioral state variations in firing rates that differ across brain regions, such that the spatial activity profile changes. The authors should consider adding a simulated test dataset for this scenario.

4. If drift correction is used as part of an automated spike sorting pipeline, one would want to be sure that the drift correction procedures do not introduce errors or otherwise degrade spike sorting performance when there is no drift in the recordings. The authors may consider adding another scenario with test signals with varying spike activity but no drift.

5. The authors report increased errors at the probe borders for both the drift estimation and signal interpolation. Border effects may be a significant source of error especially in cases when recordings span relatively small portions of the probe. Characterization of the border effect is limited, however. To what distance from the border is the effect present? What is the impact on spike sorting performance close to the border? Could the authors discuss what parts of the algorithms are most vulnerable to border effects and if their could be improvements to mitigate border effects?

Minor Comments

6. Significance statement, line 21 and Discussion, line 418: while I believe the authors did a lot of work, I do think that "exhaustive" is too strong as there is certainly room for further characterization and evaluation of drift correction procedures. I suggest that the authors tone down or rephrase.

7. Introduction, line 34: "traverse" implies an active movement and while electrodes do traverse the brain during implantation, in the context of the sentence the verb "span" may be more appropriate.

8. Line 71: for clarity, I suggest to replace "peak detection" with "spike detection", or at least make clear that the goal of the first step is spike detection using a peak detection algorithm.

9. Line 76: "blurred": I suggest to link large time windows to reduced temporal resolution of estimating drift.

10. Figure 1A: the drift shown in panel A is mainly perpendicular to the probe. Isn't drift most commonly observed in line with the probe shank? In any case, the approaches presented in the paper only correct for drift in line with the probe shank ("y axis") and as such it would be more appropriate to show that kind of drift in panel A.

11. Figure 1B: what is the purpose of the blue rectangles in the figure?

12. Figure 1C: remove the red line in the activity profile estimation plot (top left), since motion inference has not yet been performed at this stage.

13. Line 91: "to estimate the positions of the neurons" - change to "to estimate the positions of detected spikes", since no spike sorting into putative neurons has been done at this stage.

14. Page 5, equation 4: what does "ptp" stand for?

15. As I understand, the activity profile is a histogram of spiking activity over depth. What is the spatial bin size? And, how does the bin size impact drift correction performance?

16. Lines 143-150: a more detailed description of the iterative template method would be appreciated. Is "log-transformed amplitude" referring to spike peak amplitude? How many iterations are performed for each time bin? How does the iterative updating of the average template work? What is the size of the sub-blocks to refine motion estimation and are the sub-blocks overlapping?

17. Lines 158-159: for clarity, either use "spatial windows" or "spatial blocks", but not both. What "spatial prior" is applied to enforce smoothness?

18. For both the iterative template and decentralized methods, what is the impact of the size of sub-blocks (for non-rigid motion estimation) on the drift correction performance?

19. Line 161: this is the first mention of the term "drift vectors". Please add a definition of drift vectors (probably in lines 140-142).

20. Line 194: "we then selected 256 neurons" - could you explain the selection process? Perhaps, "selected" is not the correct word here? More explanation about the general procedure could be added here. As I understand, the authors sampled 256 neurons from the 13 cell models, randomly positioned each neuron along the probe shank according to some distribution, simulated a spike train for each neuron according to some rate function, convolved the spike trains with the spike waveform template of the corresponding cell model (taking into account drift) and finally sum the contributions from all cells on each electrode.

21. Could the authors conform that the "bumps" drift signal is non-rigid - the panels in figures 2 and 3 are not labeled as such. If indeed, the bumps drift signal is non-rigid, then the description in lines 212-213 need clarification, as it is not clear if cells are moved independently or coherently at either end of the probe.

22. Lines 230-231: replace italic 'x' with proper multiplication symbol.

23. In lines 232-233, it is stated that the first 60 seconds of each simulated recording is drift-free. What is the reason for this and what is the impact (if any) on the drift correction performance?

24. Lines 242-243: "From these error signals we could then visualize both the evolution of the error over time and the error profile along the depth dimension." Could you clarify how exactly these error profiles over time and depth are computed? Is this done by simple averaging?

25. Line 245: replace "ground-truth spiking activity" with "ground-truth spike timing", which seems more accurate.

26. The authors abbreviate "Ground Truth" to "GT", which is not a commonly used abbreviation and for clarity I suggest that no abbreviation is used in this case.

27. Could the authors clarify the different depth scales that are used in the figures? E.g., compare figure 5, panels A and C.

28. For motion estimation in the "bumps" drift case, one would expect that most large errors are aligned with the onset or offset of the bumps, depending on the alignment of the time bins with onsets/offsets. Have the authors looked at this?

29. Line 356, "exploding": consider using "increasing".

30. Line 356, "irregardless": either use "irrespective" or "regardless".

31. Lines 444-446, "reduces spike sorting accuracy with respect to a static recording": I believe the authors mean that in case of drift in the signals, even the best correction strategy will not recover the same spike sorting accuracy as with a static recording.

32. Lines 448-450: could the authors clarify why correcting for drifts prior to spike sorting may not be a favorable solution? Is this because of computational (in)efficiency or accuracy?

## Author Response

Response to the reviewers: A modular implementation to

handle and benchmark drift correction for high-density extracellular recordings.

Dear Editor and Reviewers, we would first like to thank the reviewers for their valuable feedback in reviewing our review titled "A modular approach to handle in-vivo drift correction for high-density extracellular recordings" . In the following we will address each referee's comments point by point, using different fonts and colors to clearly show the implemented changes: previous manuscript, the new manuscript, the reviewers' comments, and our response.

We also would like to take this opportunity to say that based on the reviewer's comments, we made two major changes to the manuscript. First, we felt the need to slightly change our title, in order to stress the point that we are benchmarking existing algorithms, not proposing a new drift correction method per se. Therefore, we propose the new title "A modular implementation to handle and benchmark drift correction for high-density extracellular recordings". The second main change was adding a new Figure 3 to highlight the main results of the motion estimation methods based on the different modes of variations of our simulations. Both reviewers found this hard to grasp and we hope that the new figure provides a better guidance to interpret the results.

Reviewer 1

Major comments

The content of the manuscript is novel and seems in large convincing. Indeed, information to assist experimentalists in setting up their spike sorting analysis pre-and post-processing stages is valuable. While I do believe this study adds to this accumulating evidence, the limitation of the study to consider data in a few selected example simulation outcomes without making a more concrete link to biology may decrease the enthusiasm a little. For this reason, below I suggest to include in the manuscript also the pipeline as a re-usable tool for researchers to adapt the example scenarios to their own needs in determining the optimal algorithms. In addition, I found the presentation of the material requires additional work in terms of the writing style, description of methodology (which is somewhat confusing and unclear due to inconsistencies and missing information) and in figure design (which are largely too small and not well designed). Some examples of this, alongside additional issues encountered, are listed below.

We would like to thank the reviewer for the comments raised. We agree that the problem with drifts in vivo is that their nature is so diverse, that it might be hard to provide a single and absolute recipe to correct for drifts in all circumstances. In this paper, we decided to choose 12 cases that, in our opinion, capture most of the complexities observed nowadays in numerous long-lasting and/or chronic recordings performed with Neuropixels probes. The complete pipeline to generate, analyse, and quantify the performance will be released jointly with the paper and should be straightforward to re-use. It relies only on open-source packages such as SpikeInterface [4] and MEArec [2].

Following the reviewer's suggestion, we attempted to improve the clarity and completeness of the manuscript, especially in the Methods section, since we observed that some details might be easily

missed in the first version of the manuscript. In addition, we modified the layout of the figures in order to make them easier to read. We hope that the manuscript, after our modifications, will be deemed suited for publication.

COG. How is a waveform defined? How is it detected that a spike (meaning putative spike, given that it could be noise?) is present on multiple channels? What is ptpi (c)? Which peaks are used in the calculation? Peak-to-trough?

The manuscript has been slightly rewritten in order to make all these concepts easier to understand. By waveforms, we refer to the spatio-temporal motif elicited, on several channels, by a single action potential of neuron. As the reviewer is guessing, such a spike can span multiple channels at once. Traditionally, most if not all spike sorting algorithms are detecting these "spikes" as threshold crossings, as we explained in the manuscript. In order to avoid putative biases due to presence of the same spike on multiple channels, peaks are detected as local minima in a small spatio-temporal region. Once a peak is detected, the corresponding waveform is cut out as a snippet of signal around the peak. From a waveform i we defined the peak-to-peak values on every channel c (ptpi (c)) as the difference between the maximum and the minimum values on each channel. Note that the peak-to-peak notation is commonly used in scientific computing (e.g., we used the numpy.ptp function, which stands for peak-to-peak). In the manuscript, we added a comment that indicates that peak-to-peak can also be referred to as peak-to-trough amplitude.

Grid: Am I correct that the described procedure is performed channel-by-channel, such that tem- plates are created for one particular channel and then all channels are convolved with these templates? I did not fully understand the logic of the algorithm. What is the (typical) range of parameter k, 10 or 10000? The use of wi conflicts with its use in COG. How is wi calculated as representation of the spike itself ? The average over wi over channels all channels? Please clarify, I think the term "spike" is used partly as spike of a neuron, partly as synonym for threshold crossing. Do fn really constitute a basis?

We agree with the reviewer that the explanation of the Grid method was unclear and we therefore modified the manuscript accordingly to make it more explicit. Indeed, the templates f⃗n are not

restricted to a single channel, but are defined on multiple channels. To construct each of them, the canonical waveform H⃗ (t) is duplicated on all channels, but with decreasing weights as explained in Equation (3). The ensemble F comprised of f⃗n∈1,...k (t) should then constitute a representative catalog

of potential templates that can be found on the probe. The intuition of the algorithm is that, in

order to get an estimated position of a given waveform, we can compute the scalar products between the waveform and a large number of "canonical" templates F , artificially created at dense, controlled positions. These scalar products can be used as "weights" in a simple barycenter formula to then infer the putative position. Typically, k has to be rather large, in the order of ≈ 20 × N where N if the number of channels in the probes. The number k will depend on the number of σ chosen (the spatial decays), and the positions p⃗n = (xn , yn ). The use of w⃗ i (t) does not seem to conflict with the COG, because we make a distinction between w⃗ i (t) (a vector, i.e. the waveforms emitted by the neuron i at

time t), and ωi,n = w⃗ i (t)f⃗n (t). ωi,n is a scalar, computed as the dot product between the waveforms

and the basis template. This is true that quite often, in the manuscript, we refer to the detected peaks

as "spikes", and this has been harmonized in the manuscript.

Mono: In Eq. 5, I assume k is ki . Is z clamped to zero for the Neuropixel probe?

In Eq 5, k is variable whose value will be optimized to find ki . While the motion correction methods used throughout the manuscript only correct for drifts along the depth of the probe, i.e., the y-axis, z

is not clamped to 0 during the estimation of the position. The monopolar approximation is the only method that can provide a z estimation, but this value is slightly more noisy (because less constrained) and not used by the current algorithms.

Motion inference: In general, I found the short descriptions hard to follow. While I understand that a complete run-down of these arguments is out-of-scope for this manuscript, I would suggest to improve clarity and precision of the write up. For example, is spike depth the same as waveform peak-to-trough as in ptp? What amplitude is taken? What is meant by bin, and how is the correlation calculated - the correlation of all bins in the shifted versus the target? How is the final drift vector shown as red line in Fig. 1C produced from the estimates? - Similarly, the motion correction section could be reworked for more clarity to improve comprehension.

We agree with the reviewer and extended the description of the motion inference methods accord- ingly. Before diving into each method details, we added a comment that explains what we refer to as spike depths: Therefore, the y estimates of the peak localization are also referred to as spike depths. We then modified the description of the two methods by adding relevant details and rephrasing por- tions of the text that could have been found confusing. For example, when describing the Iterative template method, we added a note on how spike amplitudes are calculated (i.e., the values of the trace when a putative spike is detected on the channel it was detected on). We added details on the binning procedure (The spike counts are binned over 2 s temporal windows, depths using 5 µm bins, and am- plitudes in 20 equally distributed bins), and the bin notation has been changed to shifted temporal bin of the histogram. Additionally, we added details on how the correlation measure is actually computed: The correlation is computed as the mean of the element-wise product between the shifted temporal bin and the average template. Similar updates were applied to the Decentralized description.

Line 188: What was the motivation to use layer 5 neuron models throughout the depths? Would a more diverse template library affect results? Also, it would be interesting to see the choice of templates and the corresponding effects to judge the difficulty of the problem.

We used the MEArec software to generate the simulated recordings. The software comes with L5 models 13 "pre-installed" from the Neocortical Microcircuit Collaboration Portal from the Blue Brain Project (https://bbp.epfl.ch/nmc-portal/welcome.html). While ideally a simulation would include all cell models from all layers, we believe that the 13 L5 models (9 inhibitory, 4 excitatory) capture enough morpho-electrical diversity for the purpose of generating ground-truth datasets for spike sorting evaluation. By creating such artificial recordings, we assume that other layers would share a similar variability of cell types, and thus that they would not lead to different results. However, this is a valid point, and in the same direction, one could ask whether rat/mouse/monkey/human models would lead to different outcomes. We believe that this question is out of the scope of our manuscript, but it could be investigated more in depth in later studies.

Line 192: I am not sure I understand what is meant by the drift templates.

The paragraph describing the simulation of templates with drift was modified to increase clarity. We hope that the reviewer finds our changes satisfactory.

Line 202: How were the drift amplitudes chosen for the various scenarios? Is this not a crucial parameter that may affect the benchmark, such that the drift amplitude should be varied as a parameter?

It is true that the drift amplitude can have a large role in motion correction. The value of 30 µm defined line 202 was chosen in order to be in line with drift amplitudes observed in vivo [5]. Moreover, if

we had used too large drifts, neurons at the borders of the probe would have likely been too drastically affected, leading to results hard to interpret. We agree with the reviewer that this parameter should be more carefully explored, but we made the choice to first explore the nature of the drifts, the depth distributions of neurons, and the firing rate profiles.

Line 244: How is the dispersion dealing with waveforms of one emitted spike visible on different channels ("a subset of channels")?

We made this more explicit in the manuscript. Assuming that the neuron i has fired N spikes with waveforms w⃗ 1,..N (t), we calculated the dispersion around the mean template T⃗i (t) as the standard deviation σi . The exact formula would thus be σi = std(w⃗ i − T⃗i ). However, in order to avoid gathering noise on channels where the mean template T⃗i is null, we restrict ourselves to a "subset of channels", i.e. the 5 channels where T⃗i has the largest amplitude.

Line 291: Figure 3 displays strong oscillations in the time error, especially for the COG method. How is this explainable? It seems that without those oscillations, COG would be quite on par w.r.t. errors.

The temporal oscillations of the errors simply follows the ones of the drift itself. When the drift brings cells at the border inside and outside the field of view of the probes, we can not expect the error to be null, because the motion interpolation problem is ill-constrained. And because the COG method is the worst at estimating the positions at the borders, this is the one suffering the most from these oscillations.

Figure 4: Why is there such a large jump in error at the start of the time-error plot, especially for orange?

This is a good question, which is related to the global offset of the estimated motion. While in our simulated recordings, we know that the drift is 0 over the first 60 s, the motion estimation methods are unaware of that. Thus, we can interpret motion estimation results as relative motion vectors, rather than absolute motion. To compensate for that, when comparing to the ground-truth motion, we align the estimated motion vectors to the ground-truth ones by subtracting an "optimal" offset equal to the median of the difference between the ground-truth and estimated motion. By doing so, we are ensuring that, on average, the median between the ground-truth motion and the inferred motion is null. However, this might results, as in the case highlighted by the reviewer, in an initial offset at the start of the recording.

Line 310: Is speed really an issue here? It seems that the algorithms differ at most by a factor of

4 - and parallelization could probably help as many operations are suitable for embarrassingly trivial parallelization. In addition, is this a fair comparison in terms of having optimal implementations for all methods - maybe it's just non-optimal in the way someone programmed it?

The most precise localization method, the monopolar approximation, is also the slowest. While this computational burden might not seem so relevant in the specific settings, since we use 10 minute long recordings and used a fairly large detection threshold, we found that for longer recordings with many more peaks this step does not scale very well, in the current implementation. While some optimiza- tion might be possible in the implementation, the monopolar approximation is an optimization-based method, thus not particularly well suited for GPU implementations. In the results and discussion, we therefore highlight that speed could be an issue and suggest that sub-sampling the peaks might be required for efficient performance. Nevertheless, to simplify the discussion about performance, we

decided to simplify Figure 4 and just report the total amount of time taken by each method.

Given that the manuscript can handle only a selected set of example scenarios, optimally the authors should provide their analysis pipeline in such a way that readers could - with acceptable effort - re-use it to create simulations adapted to their own scenario. I do understand this is not easy given the difficulties to package and integrate software such as Kilosort, but I would urge the authors to give it a best effort under https : //github.com/to_be_updated_af ter_review and describe very briefly the available pipeline in the manuscript.

Actually, thanks to the SpikeInterface package [4], the reproducibility and extensions of the examples highlighted in this article should be straightforward. Kilosort can be run directly in a docker container, without even a MATLAB installation. In the GitHub repository associated to the paper (that we needed to hide for the double blind revision), we are providing notebooks to generate the data, launch the pipelines, and create the figures. Following the suggestion of the reviewer, we have improved the documentation of the GitHub repository. In addition, to ease the creation of custom ground-truth recording, the documentation of MEArec have also been enhanced accordingly.

Minor comments

In general, the text reads in parts somewhat colloquial and could be polished more throughout. We did our best to modify the manuscript, trying to improve the wording and polish the text. A redundant "as one can see" in the caption of Figure 1

This has been removed

"A round of peak detection is performed" in line 73

This has been changed.

Unnecessary suggestive "important" in line 308

It has been removed.

Line 79: Instead of inverse motion signal, I assume, negative or inverted is meant.

The reviewer is right, and we changed the wording accordingly.

Figure 1. The figure is strange in the proportions of its constituent elements, especially the graphs can hardly be read even on maximum zoom levels. Panel A: please explain the panel in the caption, I was unable to interpret the figure. What are the template rows and columns? Does "Drifting positions" mean "positions after drifting", or does the figure show the dynamics during a drift? Panel B: The meaning of the blue boxes did not become clear from the description. Panel C: Why are activity profile estimation and motion inference lumped into a single (redish) box labeled motion estimation (which reads to me synonym to motion inference)? The same terminology is used in the text. I found this ambiguity a little confusing, but maybe those are established terms/hierarchies.

As suggested by the reviewer, the figure has been regenerated, and we changed the text since it was not clear enough.

Figure 2. The individual drift traces are very hard to read in this figure. The authors should try to improve their presentation of results in terms of readability, also in later Figures. The rate profiles are not sinusoidal, but seem cropped towards zero - likely due to the low likelihood to have a spike here. I would suggest to superimpose the the theoretical rate profile used for generation.

As suggested by the reviewer, the figure has been regenerated and we tried to make it easier to understand for the reader. Indeed, the rate profile is cropped towards zero to avoid periods without any spikes, and this has been better explained in the methods.

Figure 3. Parts of the figure caption seem to be in the main text (line 280-). I would suggest to improve separation between text and caption to improve readability. The overlay graphs are very difficult to read, especially for the time error. It would help to introduce time error, global error and depth error in the Methods.

The figure has been regenerated to take into account the comments from the reviewer.

Figure 4. Beside problems similar to Figure 3, the bar graph legends are nearly impossible to decipher without a good zoom functionality of the pdf reader. Figure 4C could be improved by using more tightly spaced patterns or different shades of the respective bar colours instead. Here, the legend covers a ticklabel on the y-axis.

The figure has been regenerated to take into account the comments from the reviewer.

Figure 6 uses similar colours for the categorical distinction in panels B, E, H and to map to the continuous accuracy values in panels C, F, I. Changing one of them could help to avoid potential misunderstandings. The legend for panels B, E, H seems to use internal variable names instead of more descriptive labels.

We changed the colormaps in order to avoid any confusions, and added an extra case with Kilosort 2.5 on the static recording, with motion correction turned on.

Line 303: Why is the COG (blue) excluded? Here also, iter and dec show the same trend

The CoM method has been added to the text

Line 314: I would like to better understand these yellow stripes and what is the cause here.

The yellow stripes are only occurring with the decentralized method. Since this method involves an optimization, they are likely the result of a convergence failure. In Figure 5D we added the motion vector trend at the bottom of the plot and it seems that the high-error yellow stripes are coincident with fast drift events. However, it is honestly unclear to us why they appear particularly only for one of these bumps, while it is working well almost everywhere. The authors of the decentralized method [6] confirmed that they also observed such behavior, and we therefore do not believe that it is the result of an implementation issue.

Separating figure panels with dashes seems confusing since it could also refer to a range of panels.

We regenerated all the figures and separated the panels.

The spacing between numbers and units in the text is inconsistent.

This has been corrected.

The dimensionality of some vectors was not clear to me from the text, e.g. Ti (t) on line 248. Waveform length times number of channels? In general, terms like spike, waveform, template, etc should be well defined to reduce confusion.

We thank the reviewer for the suggestion. We added a new Notation section in the Methods, in order to clarify properly the notations throughout the manuscript.

Typos

Incomplete list of typos etc:

• 10: motion correction algorithm -> a motion correction algorithm

• 18: population -> populations

• 18: via is slanted

• 20: comparisons -> comparison

• 69: as outlined as follows -> as follows

• 74: as function of -> as a function of

• 75: a time windows -> a time window

• 86: Recent work -> Recent works

• 95: allows us first to -> enables us to

• 105: an histogram -> a histogram

• 106: as function of -> as a function of

• 107: negative thresholds -> negative threshold

• 136: with cost function -> with the cost function

• 168: the the -> the

• 181: words missing? "as exponentiated negative the distance matrix"

• 183: 1e−3 -> 10−3 (?)

• 192-193: on along -> along

• 288: the errors tends -> the errors tend

• 292: misplaced comma

• 311: 4D panel -> panel 4D

• 397: Figure -> In Figure

We would like to thank the reviewer for pointing out these typos. They have all been corrected.

Reviewer 2

Major comments

My main concern is with the set of simulated signals that the authors have created and the rationale for combining variations in spatial-temporal drift and spiking activity dynamics. The 12 simulated signals are both redundant and unnecessarily complex for what the authors are trying to show and an opportunity is missed to illustrate how sensitive the tested drift correction approaches are to each of the aspects of drift and spiking activity dynamics. It would have been more informative if the impact of various aspects of drift and spiking activity on drift correct were assessed separately (unless there is a need to look at specific interactions). Relatedly, the rationale for why particular drift/spike activity variants are picked and the potential impact on the drift correction performance (given knowledge about the general drift correction approach) is not clearly explained. For example, for the drift signals, it is only stated that "Different types of drift might challenge the drift correction approaches in different ways." I assume that the choice for the zigzag and bump style drift signals is (in part) driven by the question how the drift correction algorithms handle slow or fast drift speeds and the methods/results could be presented as such. Further, it would be helpful for the authors to improve the description of the assumptions of the drift correct methods and provide more insight into the factors that impact drift correction performance. If I understand correctly, all presented drift correction procedures rely on a stable and non-uniform spatial distribution of spiking rates in the brain regions that are covered by the probe, either at the global scale (for rigid drift) or local scale (non-rigid drift). Any factors that violate these assumptions or that make it more difficult to obtain a good estimate of the spike rate distribution will likely hamper the drift correction performance. For example, a lower spiking rate or shorter time bins will reduce signal-to-noise ratio and increase variability in the estimate of the activity profile.

We thank the reviewer for the feedback. We agree that a better explanation of why we chose such variants and how the separate the distinct components of the drift signals was missing in the manuscript. We therefore extended and improved the Methods section to highlight the motivations behind our choices, and a new figure, Figure 3, that summarizes how each of these choices affects the motion estimation performance. In addition, to simplify the take home message, we re-organized the main "cases" and only kept 5 in Figure 4 (old Figure 3) and the rest as supplementary.

1. The test signals are all sampled in a single contiguous block of densely packed electrodes. With newer multi-shank probes (e.g. NP2.0) that have many more electrodes than available read-out chan- nels, experimenters often need to distribute their read-out channels across the shanks in smaller non- contiguous blocks or reduce the density at which signals are sampled. It would be useful for the authors to comment on the impact such customized channel maps may have on the drift correction performance.

We agree with the reviewer that the effect of specific electrode configurations and densities could play an important role in motion correction. We therefore added a paragraph in the Discussion to cover this topic.

2. The authors simulated the spike trains of 250 neurons with locations randomly drawn from a uniform or bimodal distribution and either constant or sine-modulated firing rate.

2a. First a note that, as far as I understand, a uniform activity profile is not desirable for drift correction. The authors probably got away with using a uniform location distribution because the number of neurons per mm along the shank is sufficiently sparse, which by chance resulted in a non- uniform activity profile.

The reviewer is right. A uniform activity profile, assuming all cells would fire similarly (at same rates, and with same peak amplitudes) would clearly be not optimal for motion estimation. Indeed, the idea behind the motion correction algorithms is to find some key "anchors" in the activity profile such that they could be used to estimate the motion. If the activity profile were flat along the depth axis, then it motion estimation would be doomed. However, even with a uniform distribution of the positions, we do not see a flat activity profile for two reasons: first, the number of neurons is sparse enough so that the distribution is not really uniform; second, and more importantly, because cells have various morphologies and orientations, which results in different spike amplitudes. In the paper (same as in Kilosort ), the activity profile is built only considering large peaks 10 larger than the noise levels. Since not all spikes are fulfilling this property, the activity profile is non-homogeneous enough to allow motion estimation algorithms to work well.

2b. As rationale for using uniform and bimodal location distributions for neuron location, the authors state that the distribution of neurons along the probe "might result in portions of the probe experiencing lower spiking activity". This requires further explanation, also because it appears to conflate the issues of spiking rate and spatial distribution of activity. For global (using all electrodes) correction of rigid drift, low spiking activity in portions of the probe contributes to a non-uniform activity profile and may thus be beneficial. On the other hand, for algorithms that use smaller portions of the probe to perform local correction of non-rigid drift, low spiking activity in those portions will pose a challenge.

We agree with the reviewer and this is why we wanted to investigate if the performance of the algorithms would depend on the distribution of the neurons. We tried to extend the motivation of our choice in the Methods. In addition, panel B of the newly added Figure 3 shows that the overall performance of motion estimation is only slightly worse than the uniform case.

2c. As rationale for using constant and sine-modulated spike rates, the authors state that "drift cor- rection methods may assume stationary firing rates". Again, more explanation is needed on why drift correction method assume stationary firing rates. Additionally, the sine-modulated test signal may test the impact of low spiking rates rather than non-stationarity.

To motivate the choices we made for the drift signals/distributions of neurons/firing rates that we tested, we added a new Figure 3 in the manuscript, summarizing the global effect of these choices on the errors performed when estimating the motion. In addition, we extended the explanation in the text to make our statement more explicit. The key point is that if cells are firing in a non-stationary manner, then the activity profile over time might change, regardless of the changes in the neuron positions.

2d. In the results, no actual comparison is performed for the two neuron location distributions or for the two firing rate scenarios.

We adapted the choices of our examples, in order to explain more in depth the various levels of complexity that have been considered. To quantify this, we added a new Figure 3 at the beginning of the results section, summarizing the global errors performed, on averaged, depending on the different aspects of our synthetic drifts.

3. One challenge for drift correction procedures are behavioral state variations in firing rates that differ across brain regions, such that the spatial activity profile changes. The authors should consider adding a simulated test dataset for this scenario.

We agree with the reviewer that this should be a very interesting point to test. However, since

there are no clear models on brain states (sleep, awake, ripples, ...) and/or since it might depend a lot on the animals and the state of the animal, we decided to leave this point aside in the current study. Nevertheless, we added a comment about brain states in the Discussion section.

4. If drift correction is used as part of an automated spike sorting pipeline, one would want to be sure that the drift correction procedures do not introduce errors or otherwise degrade spike sorting performance when there is no drift in the recordings. The authors may consider adding another scenario with test signals with varying spike activity but no drift.

We would like to thank the reviewer for the suggestion. To answer this particular point, the last figure of the results (Figure 7) has been updated, adding an extra case displaying the performance of Kilosort when launched on the static recording, with motion correction activated. As one can notice, the performance is barely affected. We added relevant comments in the text.

5. The authors report increased errors at the probe borders for both the drift estimation and signal interpolation. Border effects may be a significant source of error especially in cases when recordings span relatively small portions of the probe. Characterization of the border effect is limited, however. To what distance from the border is the effect present? What is the impact on spike sorting performance close to the border? Could the authors discuss what parts of the algorithms are most vulnerable to border effects and if their could be improvements to mitigate border effects?

The exact distance from the probe borders at which the performance of motion correction pipelines will start to deteriorate depends on the parameters (mostly on the spatial bin size used to compute the activity histogram as function of the depth, here 5 µm), the distribution of the neurons, and the amount of drift. While both motion estimation and motion interpolation are affected, the interpolation of the traces is clearly the step suffering the most from this border effect, since for movements that will go outside of the probe, the traces can only be interpolated given the channels within the layout of the probes, and thus information is missing. Ideally, one should try to exclude the channels at the border from downstream analysis, because some of their statistical properties, such as noise levels, can be impacted and affect spike sorting algorithms. In SpikeInterface , we implemented this as an interpolation option so that users can set the border_option='remove_channels' to remove the border channels based on the maximum estimated drift. For the datasets used in this manuscript, one can see from the new Figure 3 that errors tend to increase around 100 µm from the border, but again this threshold will depend on the motion itself. We highlighted this option in the "Drift correction with SpikeInterface " section.

Minor comments

6. Significance statement, line 21 and Discussion, line 418: while I believe the authors did a lot of work, I do think that "exhaustive" is too strong as there is certainly room for further characterization and evaluation of drift correction procedures. I suggest that the authors tone down or rephrase.

We removed the word "exhaustive" as suggested by the reviewer.

7. Introduction, line 34: "traverse" implies an active movement and while electrodes do traverse the brain during implantation, in the context of the sentence the verb "span" may be more appropriate.

We changed the wording according to the reviewer's suggestion.

8. Line 71: for clarity, I suggest to replace "peak detection" with "spike detection", or at least make clear that the goal of the first step is spike detection using a peak detection algorithm.

This has been added, as suggested by the reviewer.

9. Line 76: "blurred": I suggest to link large time windows to reduced temporal resolution of estimating drift.

We thank the reviewer for this interesting suggestion, but we are not sure that it would entirely solve the problem. This is because the assumption of the different methods is that the activity profile is stationary within a temporal window. Having large windows, even if they are overlapping, would still suffer from the same blurring problem, but yield a higher temporal resolution in the motion estimation (which could also be achieved by temporal interpolation).

10. Figure 1A: the drift shown in panel A is mainly perpendicular to the probe. Isn't drift most commonly observed in line with the probe shank? In any case, the approaches presented in the paper only correct for drift in line with the probe shank ("y axis") and as such it would be more appropriate to show that kind of drift in panel A.

In principle, drift could appear in any direction, but we agree with the reviewer that most of the drift will be observed as being perpendicular to the probe. Nevertheless, the schematic in Figure 1A is meant to be purely illustrative and is adapted from another contribution [3]. In the Methods section where we describe the different algorithms, we further highlighted that drift is estimated and corrected only along the y-axis (depth).

11. Figure 1B: what is the purpose of the blue rectangles in the figure?

The blue rectangle shows part of the recording where spontaneous drifts are clearly visible, in a non- homogeneous/non-rigid way. This has been added in the legend.

12. Figure 1C: remove the red line in the activity profile estimation plot (top left), since motion inference has not yet been performed at this stage.

This has been corrected and the figure has been regenerated.

13. Line 91: "to estimate the positions of the neurons" - change to "to estimate the positions of detected spikes", since no spike sorting into putative neurons has been done at this stage.

This has been modified as suggested.

14. Page 5, equation 4: what does "ptp" stand for?

"ptp" stands for "peak to peak" and it has been better explained in the manuscript.

15. As I understand, the activity profile is a histogram of spiking activity over depth. What is the spatial bin size? And, how does the bin size impact drift correction performance?

The default bin size used in the manuscript is 5 µm, same as in Kilosort . It has been added in the Methods of the paper. However, we did not explore thoroughly the influence of this parameter

on the motion estimation. Decreasing it could enhance the spatial resolution, at the risk of ending up with spatial bins with only few or even no spike at all. On the other hand, large bin might "blur" the estimated motion, and underestimate the motion vectors.

16. Lines 143-150: a more detailed description of the iterative template method would be appreciated. Is "log-transformed amplitude" referring to spike peak amplitude? How many iterations are performed for each time bin? How does the iterative updating of the average template work? What is the size of the sub-blocks to refine motion estimation and are the sub-blocks overlapping?

We extended the explanation of the different the motion estimation and interpolation methods.

17. Lines 158-159: for clarity, either use "spatial windows" or "spatial blocks", but not both. What

"spatial prior" is applied to enforce smoothness?

This has been corrected and we are now only using the term "spatial blocks". Regarding the "spatial prior", we refer to [7] for more details.

18. For both the iterative template and decentralized methods, what is the impact of the size of sub-blocks

(for non-rigid motion estimation) on the drift correction performance?

We did not explore that parameter, but we agree with the reviewer that it should be looked at more in depth in further studies. We added a sentence in the Discussion to highlight the fact that in the current study, we did not explored the (large) parameter space for the individual pipelines: Finally, in this work we did not explore the relatively large parameter space associated to the different methods that we used, but we rather chose default parameters suggested by the original implementations. Future improvements could target fine-tuning the parameters to further improve the performance of motion correction methods.

19. Line 161: this is the first mention of the term "drift vectors". Please add a definition of drift vectors (probably in lines 140-142).

A definition has been added, as suggested.

20. Line 194: "we then selected 256 neurons" - could you explain the selection process? Perhaps, "selected" is not the correct word here? More explanation about the general procedure could be added here. As I understand, the authors sampled 256 neurons from the 13 cell models, randomly positioned each neuron along the probe shank according to some distribution, simulated a spike train for each neuron according to some rate function, convolved the spike trains with the spike waveform template of the corresponding cell model (taking into account drift) and finally sum the contributions from all cells on each electrode.

Actually, this is indeed a selection process as stated in the manuscript. We initially generated 100 templates for every cell models (13 distinct L5 cells), leading to a total of 13 × 100 = 1300 templates. All these templates are unique (and not simply translated version of each others), because every cell has a unique orientation, position, depth and cell type. Given all these templates, for each recording we selected a subset of them, either randomly or by taking their depths into account (for the bimodal depth distribution). Once these cells have been selected, we then generated the spike trains (either as homogeneous Poisson processes or non-homogeneous ones with sine modulation), and convolved them with the templates, adding some amplitude modulation in the process to model biophysical waveform variability.

21. Could the authors confirm that the "bumps" drift signal is non-rigid - the panels in figures 2 and

3 are not labeled as such. If indeed, the bumps drift signal is non-rigid, then the description in lines

212-213 need clarification, as it is not clear if cells are moved independently or coherently at either end of the probe.

We confirm that the Bumps case is non rigid. The Methods section has been rewritten in order to make this more explicit. In addition, we added a "Drift gradient" illustration in Figure 2 and Figure S1 to all non-rigid cases.

22. Lines 230-231: replace italic 'x' with proper multiplication symbol.

This has been modified

23. In lines 232-233, it is stated that the first 60 seconds of each simulated recording is drift-free. What is the reason for this and what is the impact (if any) on the drift correction performance?

The motivation for this 'drift free" period of 60s is that it could be use as a sanity check to assess that all the random selections performed when generating the drifting signals where not creating some subtle differences between the static and the drifting signals (with respect to the selected cells, spike trains, noise in the peak amplitudes, ...). We did not check if such a period had an impact on the drift correction performances, but we do not think that this should affect the motion correction results.

24. Lines 242-243: "From these error signals we could then visualize both the evolution of the error over time and the error profile along the depth dimension." Could you clarify how exactly these error profiles over time and depth are computed? Is this done by simple averaging?

Yes, we are simply performing either a time or a depth average and this process is now more explicit in the manuscript.

25. Line 245: replace "ground-truth spiking activity" with "ground-truth spike timing", which seems more accurate.

This has been modified.

26. The authors abbreviate "Ground Truth" to "GT", which is not a commonly used abbreviation and for clarity I suggest that no abbreviation is used in this case.

We decided to keep the naming, but added an explanation in the new Notations section of the methods.

27. Could the authors clarify the different depth scales that are used in the figures? E.g., compare figure 5, panels A and C.

In Figure 6 (old Figure 5), the probes are cropped version of the probes. We added a comment about this in the caption to make this more explicitly.

28. For motion estimation in the "bumps" drift case, one would expect that most large errors are aligned with the onset or offset of the bumps, depending on the alignment of the time bins with onsets/offsets. Have the authors looked at this?

We would like to thank the reviewer for the suggestion. Figure 5 has been regenerated with an added panel of the added motion, so that it is easier for the reader to appreciate how the errors are aligned with the drift onsets.

29. Line 356, "exploding": consider using "increasing".

This has been modified as suggested.

30. Line 356, "irregardless": either use "irrespective" or "regardless".

This has been modified as suggested.

31. Lines 444-446, "reduces spike sorting accuracy with respect to a static recording": I believe the authors mean that in case of drift in the signals, even the best correction strategy will not recover the same spike sorting accuracy as with a static recording.

Yes, this is exactly what we meant and this has been better explained in the revised manuscript.

32. Lines 448-450: could the authors clarify why correcting for drifts prior to spike sorting may not be a favorable solution? Is this because of computational (in)efficiency or accuracy?

We tried to clarify the text accordingly. The point we want to make is that interpolating the traces is distorting the waveforms, but also the noise structure. What we meant is that maybe it would be better to use such motion signals to distort the templates during the template matching procedure instead of the entire traces. By doing so, only the templates will be modified, and no side-effects on the noise levels will be observed. Note that this approach has been adopted in a recent contribution [1], which is now cited in the manuscript.

References

[1] J. Boussard, C. Windolf, C. Hurwitz, H. D. Lee, H. Yu, O. Winter, and L. Paninski. Dartsort: A

modular drift tracking spike sorter for high-density multi-electrode probes. bioRxiv, pages 2023-08,

2023.

[2] A. P. Buccino and G. T. Einevoll. Mearec: a fast and customizable testbench simulator for ground- truth extracellular spiking activity. Neuroinformatics, pages 1-20, 2020.

[3] A. P. Buccino, S. Garcia, and P. Yger. Spike sorting: new trends and challenges of the era of high-density probes. Progress in Biomedical Engineering, 4(2):022005, 2022.

[4] A. P. Buccino, C. L. Hurwitz, S. Garcia, J. Magland, J. H. Siegle, R. Hurwitz, and M. H. Hennig.

Spikeinterface, a unified framework for spike sorting. Elife, 9:e61834, 2020.

[5] N. A. Steinmetz, C. Aydin, A. Lebedeva, M. Okun, M. Pachitariu, M. Bauza, M. Beau, J. Bhagat, C. Böhm, M. Broux, et al. Neuropixels 2.0: A miniaturized high-density probe for stable, long-term brain recordings. Science, 372(6539), 2021.

[6] E. Varol, J. Boussard, N. Dethe, O. Winter, A. Urai, T. I. B. Laboratory, A. Churchland, N. Stein- metz, and L. Paninski. Decentralized motion inference and registration of neuropixel data. In ICASSP 2021-2021 IEEE International Conference on Acoustics, Speech and Signal Processing (ICASSP), pages 1085-1089. IEEE, 2021.

[7[ C. Windolf, A. C. Paulk, Y. Kfir, E. Trautmann, D. Meszena, W. M uiioz, I. Caprara, M. Jamali, J. Boussard, Z. M. Williams, et al. Robust online multiband drift estimation in electrophysiol­ ogy data. In ICASSP 2023-2023 IEEE International Conference on Acoustics, Speech and Signal Processing (ICASSP), pages 1-5. IEEE, 2023.
